# Active Expression of Genes for Protein Modification Enzymes in Habu Venom Glands

**DOI:** 10.3390/toxins14050300

**Published:** 2022-04-22

**Authors:** Akiko Isomoto, Eiichi Shoguchi, Kanako Hisata, Jun Inoue, Yinrui Sun, Kenji Inaba, Noriyuki Satoh, Tomohisa Ogawa, Hiroki Shibata

**Affiliations:** 1 Division of Genomics, Medical Institute of Bioregulation, Kyushu University, Fukuoka 812-8582, Japan; syr0221@gen.kyushu-u.ac.jp; 2 Department of Biological Chemistry, Graduate School of Agricultural Science, Tohoku University, Sendai 980-8572, Japan; 3 Marine Genomics Unit, Okinawa Institute of Science, Technology Graduate University, Onna 904-0495, Japan; eiichi@oist.jp (E.S.); kanako@oist.jp (K.H.); jun.inoue@oist.jp (J.I.); norisky@oist.jp (N.S.); 4 Institute of Multidisciplinary Research for Advanced Material, Tohoku University, Sendai 980-8577, Japan; kenji.inaba.a1@tohoku.ac.jp; 5 Department of Biomolecular Science, Graduate School of Life Sciences, Tohoku University, Sendai 980-8577, Japan

**Keywords:** venom protein modification enzyme, disulfide-bond, PDI, venom protein folding, *P4HB*, *PDIA3*

## Abstract

Genes encoding snake venom toxins have been studied extensively. However, genes involved in the modification and functioning of venom proteins are little known. *Protobothrops* is a genus of pit vipers, which are venomous and inhabit the Nansei (Southwest) islands of Japan, Taiwan China, Vietnam, Thailand, Myanmar, Nepal, Bhutan, and India. Our previous study decoded the genome of *Protobothrops flavoviridis*, a species endemic to the Nansei Islands, Japan, and revealed unique evolutionary processes of some venom genes. In this study, we analyzed genes that are highly expressed in venom glands to survey genes for candidate enzymes or chaperone proteins involved in toxin folding and modification. We found that, in addition to genes that encode venom proteins and ribosomal proteins, genes that encode protein disulfide isomerase (PDI) family members (orthologs of human *P4HB* and *PDIA3*), Selenoprotein M (*SELENOM*), and Calreticulin (*CALR*) are highly expressed in venom glands. Since these enzymes or chaperones are involved in protein modification and potentially possess protein folding functions, we propose that *P4HB*, *SELENOM*, *CALR*, and *PDIA3* encode candidate enzymes or chaperones to confer toxic functions upon the venom transcriptome.

## 1. Introduction

Several crotalid snakes inhabit the Nansei (southwestern) Islands of Japan (Amami-Oshima, Tokunoshima, Okinawa, and Sakishima) and Taiwan [1]. They include *Protobothrops flavoviridis* (Amami-Oshima, Tokunoshima, and Okinawa islands), *Ovophis okinavensis* (Amami-Oshima, Tokunoshima, and Okinawa islands), *P. elegans* (Ishigaki and Iriomote islands of the Sakishima Islands), *P. mucrosquamatus* (Taiwan), and *Trimeresurus stejnegeri* (Taiwan) (The Reptile Database: http://www.reptile-database.org/ (accessed on 31 March 2019)). From the point of view of snake envenomation in Japan, the habu, *P. flavoviridis*, is the greatest threat to people, with more than 1000 people bitten per year, and a fatality rate of 1% [2].

To clarify snake venom repertoires, as well as the evolution and physiological function of these venoms, many proteomic and transcriptomic studies have been performed [3,4,5]. These have revealed that snake venom proteins are produced as isoforms with various physiological functions and considerable inter- and intraspecific variation. This diversification of venom proteins may be caused by various molecular evolutionary mechanisms, including the accelerated evolution of venom genes [6,7,8,9] and alternative splicing of transcripts [10].

Since genomic information is essential for further studies on snake venom, we decoded the *P. flavoviridis* genome in 2018 [9]. In that study, we identified 60 venom genes belonging to 18 protein families. Interestingly, those genes arose through multiple duplication processes, resulting in many paralogs for each gene [9]. The habu genome analysis also demonstrated that six protein families (metalloproteinase (MP), serine protease (SP), C-type lectin-like proteins (CTLP), phospholipase A_2_ (PLA_2_), three-finger toxins (3FTX), and cysteine-rich secretory proteins (CRISP)) show accelerated evolution [9].

However, there are many questions to be answered in order to understand snake venom evolution, physiology, and pharmacology. One important question concerns how venom proteins are processed post-translationally to acquire their toxic functions. For example, one of the salient characteristics of venom proteins and peptides from venomous snakes, scorpions, spiders, and sea snails is that they are rich in disulfide bonds that stabilize their 3D structures [11,12]. PLA_2_s, which are major habu venom components, contain seven disulfide bonds. Type III svMPs, such as flavorase, HR1A, HR1B, and HVI1, possess 17 disulfide bonds, and a metalloprotease domain with disintegrin-like and cysteine (Cys)-rich domains at the C-terminus. In addition, CTLP, 3FTXs, and CRISP family proteins also contain four or more disulfide bonds. Furthermore, a certain type of CTLP forms an interchain disulfide bond with type III MPs, resulting in type IV MPs [13]. Thus, most *P. flavoviridis* snake venom proteins are Cys-rich proteins. It is thought that venom proteins pass through several modification processes to assume fully functional forms.

As mentioned above, although studies of venom transcriptomes have been conducted for many venomous snakes [3,4,5], there is little information on non-toxin transcripts expressed in venom glands. Moreover, there are even fewer reports focused on genes for enzymes that modify protein structure. Rokyta et al. (2012) [14] carried out transcriptomic analyses of venom glands of the eastern diamondback rattlesnake (*Crotalus adamanteus*) and found that genes for protein disulfide isomerases (PDIs) are highly expressed in the venom glands. In addition, Luna et al. (2013) [15] carried out proteomic analysis of the venom glands of *Bothrops jararaca* and found that proteins from the endoplasmic reticulum (ER), such as PDI and GPR78, and cytoplasmic proteins, such as 40S ribosomal protein, are enriched in venom glands. We expect that more genes involved in protein-modification functions are also highly expressed in venom glands of habus. Thus, to identify genes for candidate enzymes or chaperones of venom protein modification in *P. flavoviridis*, we carried out comprehensive transcriptomic analyses of venom glands and other organs. Here, we propose *P4HB, PDIA3*, *SELENOM*, and *CALR* as candidate enzyme genes for regulating/modifying venom proteins, since these genes are suggested to be strongly associated with the oxidative folding of venom proteins with multiple disulfide bonds.

## 2. Results

### 2.1. Identification of Candidate Genes for Modification, Folding, and Quality Control of Venom Proteins

In the *Protobothrops flavoviridis* whole-genome decoding project, we carried out thorough transcriptomic analyses of 20 organs and tissues of an adult snake, which included the venom gland (sample name: venom gland-specimen#1), venom fang-forming tissue, lung, liver, kidney, small intestine, colon, stomach, pancreas, heart, masseter muscle, brain (brain-specimen#1), eye, nose, pit organ, spleen, and ovary (accession # DRA006600, see Supplementary Table S2 of Shibata et al. [9]). In addition, we also analyzed mRNAs of the venom gland (venom gland-specimen#2), brain (brain-specimen#2), and accessory gland of another adult, as well as the neonate venom gland, and fetal fibroblasts from a neonate. We carried out comprehensive comparative analyses of these data to identify genes for enzymes with protein modification functions. All mRNAs were identified and characterized from habu whole-genomic information [9]. We expected that genes for venom-protein modification enzymes would also be more highly expressed in venom glands than in other organs and tissues, such as in digestive organs.

We first identified the 50 genes that are most highly expressed in habu venom glands (Table 1 and Supplementary Table S1). Based on the number of transcripts per million reads (TPM), there were no differences between the expression profiles of the two brains. The two venom glands also showed similar profiles of highly expressed genes. However, there were different numbers of venom protein genes in these two specimens (Table 1 and Supplementary Table S1). The venom gland from the second adult specimen included more venom protein genes (19 genes) (Table 1) than that from the first specimen (12 genes) (Supplementary Table S1), suggesting that the second specimen (Table 1) may have been actively synthesizing venom (Supplementary Table S1). Therefore, we profiled the expression of protein modification enzymes in the venom gland from the second specimen as a main focus (Table 1).

Table 1 shows the 50 most highly expressed genes in the venom gland from the second specimen. Fourteen of the top sixteen genes were categorized as venom protein genes: including bradykinin-potentiating and C-type natriuretic peptides (*CNP01*), *svPLA2-02* and *-03*, *svMP06*, *svPLA2-01*, *-07*, and *-08*, *svMP07* and *-08*, *svCTLP06*, and *-07*, *svMP04*, *svCLTP08*, *svMP05*, *svSP04* and *LAAO01* (Table 1), which were all the same as in the venom gland from the other adult specimen except for *svSP04* and *LAAO01* (Supplementary Table S1). Moreover, other venom protein genes, such as *CRISP01* and *02*, *svSP11*, *svMP09* and *-10*, *svMP01*, and *sv5Nase*, were also included in the top 50 genes of only the second adult specimen (Table 1).

**Figure 1:**
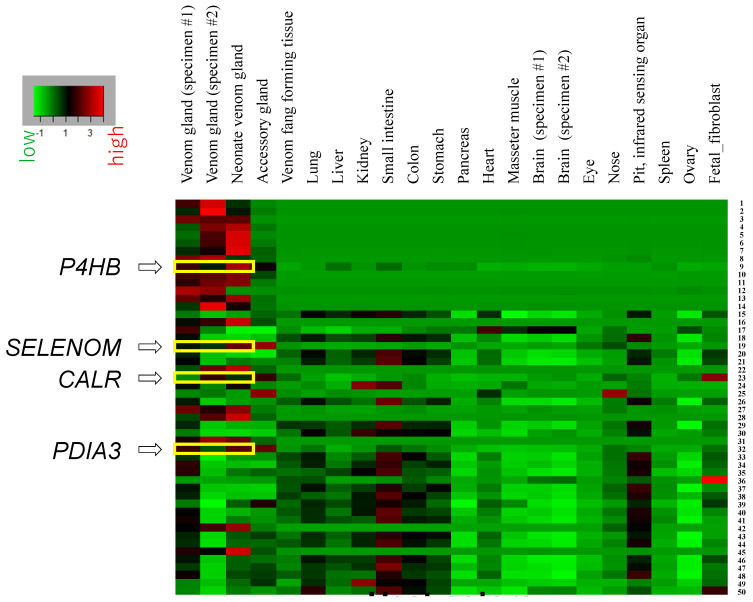
Heat map representing genes predominantly expressed in habu venom glands and other organs. Numbers for each gene correspond to genes in Table 1. Genes for candidate modification enzymes or chaperones related to protein folding are indicated with yellow boxes.

Interestingly, we found that a gene for protein disulfide isomerase (*PDI*), also known as the beta-subunit of prolyl 4-hydroxylase (*P4HB*), was the ninth most highly expressed gene in one adult venom gland (Table 1) and the sixth most highly expressed in the other specimen (Supplementary Table S1). In addition, genes for Selenoprotein M, Calreticulin, and protein disulfide isomerase A3 (*SELENOM*, *CALR* and *PDIA3*) were ranked as the 19th, 23rd, and 31st most highly expressed genes, respectively (Table 1). These four genes are likely involved in the folding and quality control of venom proteins. The high expression profiles of these genes were further confirmed with another venom gland from the first adult specimen and in neonate venom glands (Figure 1 and Table 2). The high expression of *PDI* genes in habu venom glands was consistent with the *PDI* expression profile of *Crotalus adamanteus* [14], suggesting that snake venom glands actively express genes not only for toxic proteins, but also for enzymes that fold and modify proteins in other vertebrate tissues.

Table 1 also shows that many genes associated with translation and protein biosynthesis, including 40S and 60S ribosomal proteins, elongation factor 1 (EF1)-alpha 1 and EF1-beta are highly expressed in habu venom glands, an observation reported previously [14,15].

### 2.2. Expression Profiles of Highly Active Genes, P4HB, SELENOM, CALR, and PDIA3, Are Specific to Venom Glands

Our analysis confirmed the highly active expression of *P4HB, SELENOM, CALR*, and *PDIA3* genes in habu venom glands. However, since *P4HB, SELENOM, CALR*, or *PDIA3* participate in protein folding and modification as part of the ER chaperone system, the active expression of these genes in the venom gland does not necessarily imply the lower expression of these genes in other organs and tissues. To examine the expression profiles of these genes in various organs, we compared the expression levels of the top 50 venom gland genes in other tissues (Figure 1). The expression profiles of those 50 genes were shared only by adult and neonate venom glands, but not by other tissues, clearly indicating that the venom gland gene expression profile is unique and specific and is quite distinct from those of other tissues (Figure 1 and Table 2). Interestingly, the expression profile of the neonate venom gland was similar to that of two adult venom glands. This similarity is especially clear for chaperone proteins, such as PDIs, Selenoprotein M, and Calreticulin (Figure 1). In the 50 most highly expressed venom gland genes, another common expression profile was seen among the lungs, kidneys, small intestine, colon, stomach, and pit organ (Figure 1). In the case of genes encoding ribosomal proteins, high expression profiles were observed not only in venom glands, but also in the lungs and small intestine.

### 2.3. Characterization of Genes for PDI Family Proteins and PDI-Related Proteins in Habu Venom Glands

**Figure 2:**
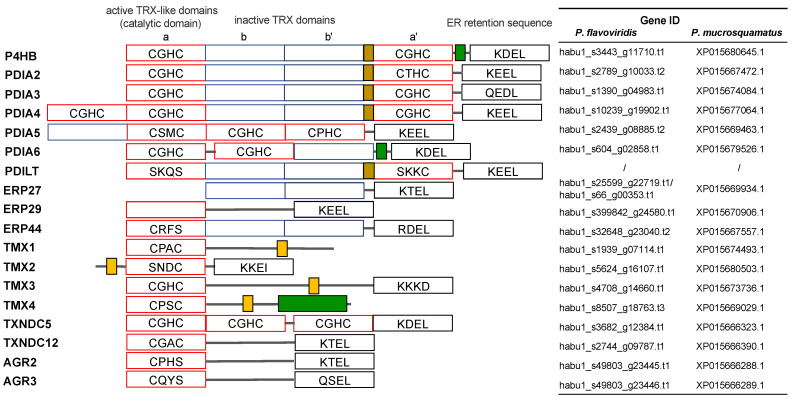
Schematic representation of *PDI* gene family members in humans and their domain composition. Catalytically active domains (**a**) and (**a’**) are shown in red; inactive domains in light blue (**b**) and dark blue (**b’**); Asp/Glu rich Ca^2+^-binding domains are designated in green; orange represents transmembrane domains; x-linker regions (brown); COOH terminal ER retention sequences (white). Habu *PDI* family Gene IDs for *P. flavoviridis* and *P. mucrosquamatus* that are orthologs of human *PDI* family genes, are shown on the right side. This figure on the left side was adapted and modified with permission from [17] (under the terms of the Creative Commons Attribution License).

**Figure 3:**
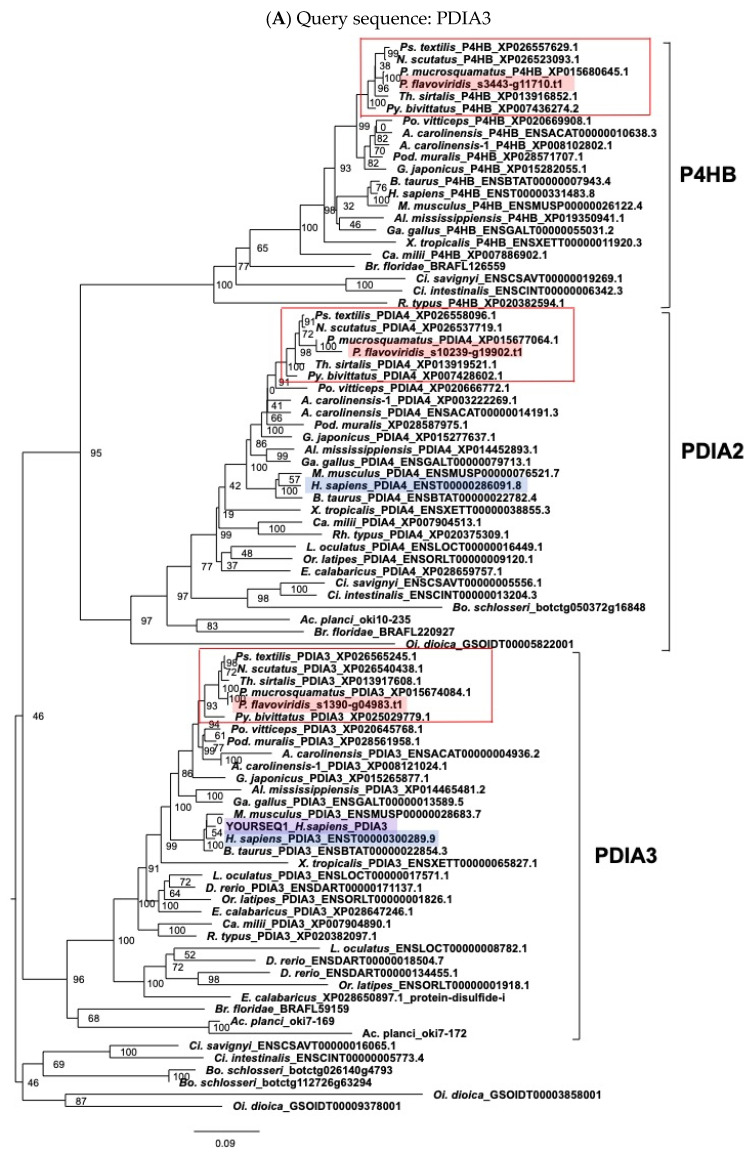

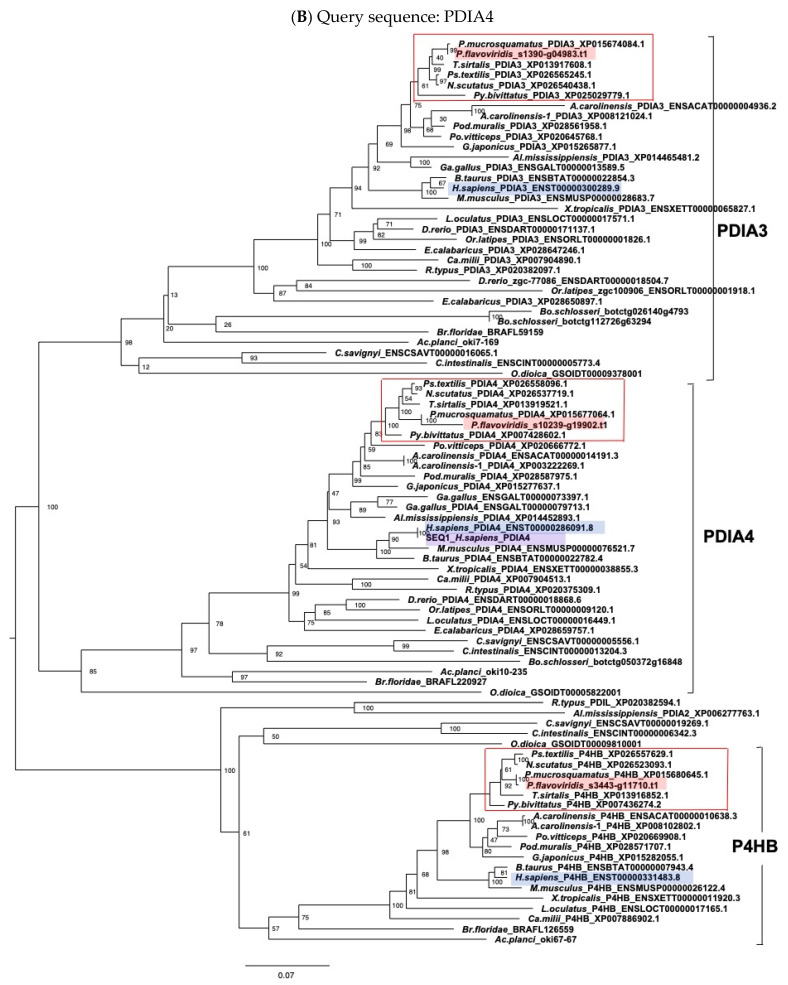

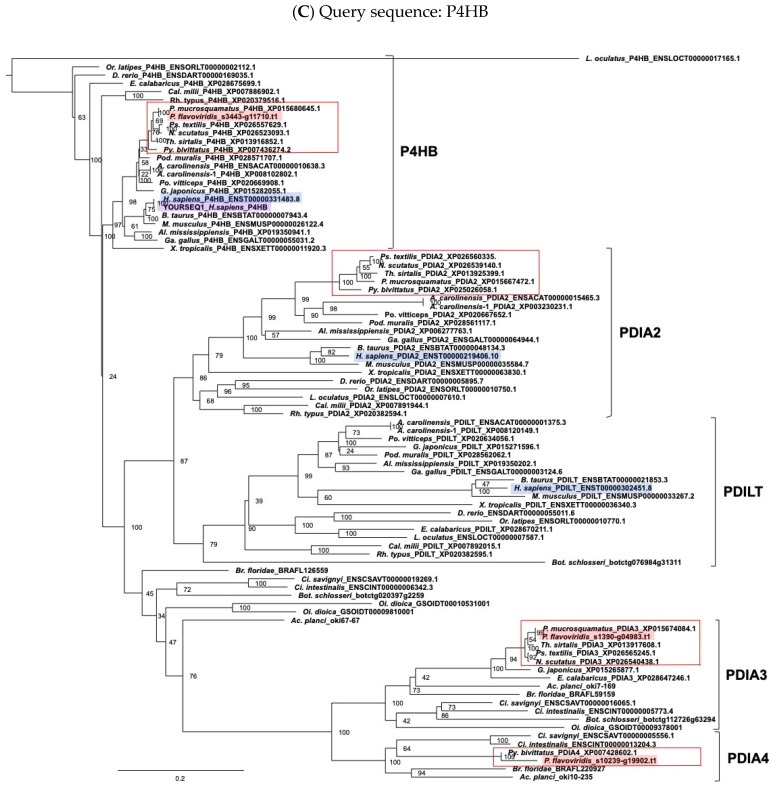

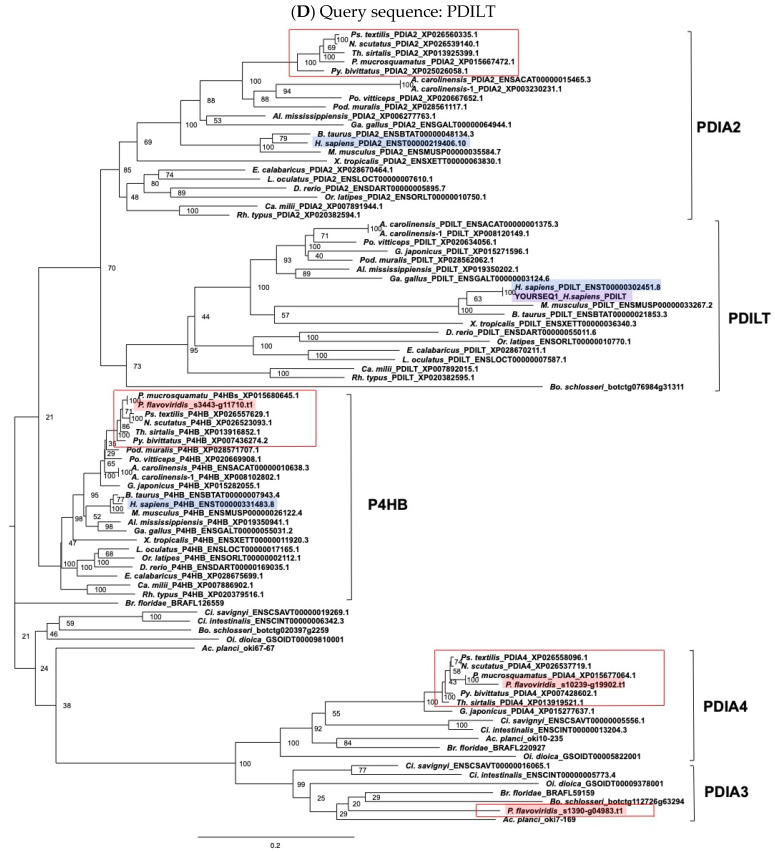

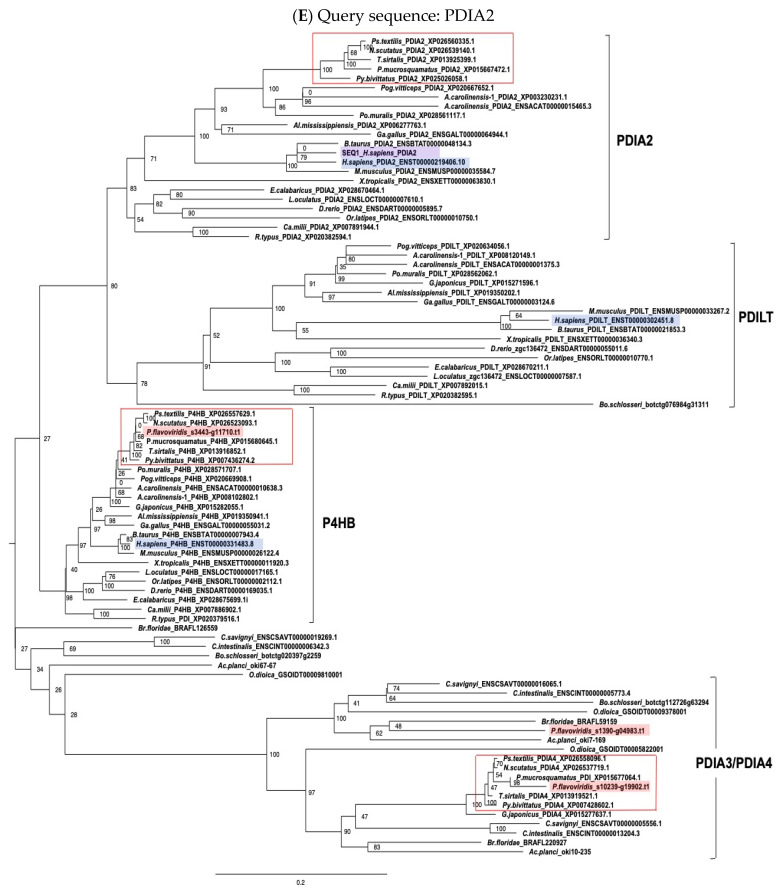
Molecular phylogenetic trees of *protein disulfide isomerases (PDIs)*. These trees were estimated using ORTHOSCOPE with human *PDIA3* (**A**), *PDIA4* (**B**), *P4HB* (**C**), *PDILT* (**D**), and *PDIA2* (**E**) as query sequences (highlighted in purple-colored), respectively. Sequences highlighted in blue indicate human orthologs. Those highlighted in pink represent habu (*P. flavoviridis*) orthologs, and those in red boxes represent other snake species. Values beside branches represent the percentages of times that nodes were supported in 100 bootstrap pseudoreplications implemented in ORTHOSCOPE. The scale bar indicates an evolutionary distance of 0.2 substitutions per position.

**Figure 4:**
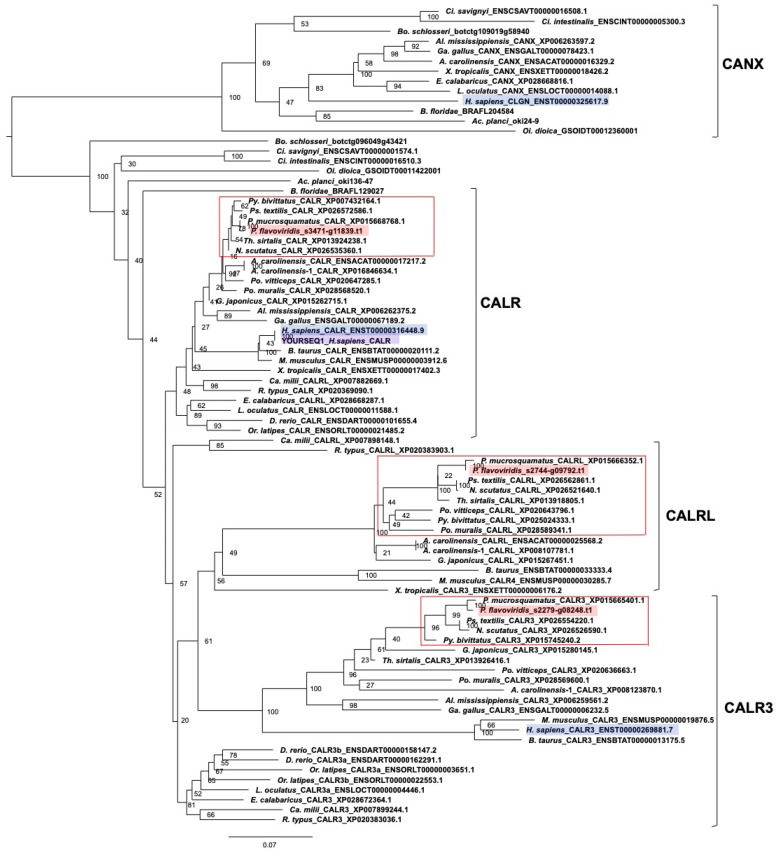
Molecular phylogenetic tree of *CALR*. This tree was estimated using ORTHOSCOPE with human *CALR* as the query sequence (highlighted in purple-colored). The tree includes *CALR, CALR-like, CALR3* and *CANX* clades. Sequences highlighted in blue indicate human orthologs. Those highlighted in pink represent habu (*P. flavoviridis*) orthologs, and those in red boxes represent other snake species. Values beside branches represent the percentages of times that nodes were supported in 100 bootstrap pseudoreplications implemented in ORTHOSCOPE. The scale bar indicates an evolutionary distance of 0.07 substitutions per position.

As already mentioned, major venom proteins, such as PLA_2_, MPs, CTLP, and 3FTXs contain many disulfide bonds. Genes for PDI are ultra-highly expressed in venom glands. Therefore, to identify and characterize genes for PDI family members (Figure 2) in the habu genome, we carried out molecular phylogenetic analysis, taking advantage of the habu whole genome. We constructed molecular phylogenetic trees using ORTHOSCOPE, a species tree-based ortholog group identification tool [16], with human *PDIA3, PDIA4, P4HB, PDILT, PDIA2* (Figure 3), *PDIA5* (Supplementary Figure S1), and *PDIA6* (Supplementary Figure S2) as query sequences for each. In addition, genes for other PDI family-related proteins were also analyzed using ORTHOSCOPE to identify orthologs in *P. flavoviridis.* These PDI family-related proteins include oxidoreductases, such as ERP27 (Supplementary Figure S3), ERP29 (Supplementary Figure S4), and ERP44 (Supplementary Figure S5), anterior gradient protein 2 (AGR2) (Supplementary Figure S6), anterior gradient protein 3 (AGR3) (Supplementary Figure S7), and transmembrane oxidoreductases, such as TMX1, TMX2, TMX3, and TMX4 (Supplementary Figures S8–S11), CALR members (Figure 4), TXNDC5 (Figure S12), and TXNDC12 (Supplementary Figure S13), redox partner proteins of PDIs, and selenoproteins. Genes identified in the habu (*P. flavoviridis*) genome and in transcriptomes of a related snake (*P. mucrosquamatus*) are summarized in Figure 2 and Table 2.

(a) PDI family

Molecular phylogenetic trees of *PDI* family genes were estimated with ORTHOSCOPE using human *PDIA3* (Figure 3A), *PDIA4* (Figure 3B), *P4HB* (Figure 3C), *PDILT* (Figure 3D), and *PDIA2* (Figure 3E) as query sequences, showing that these trees constitute discrete clades of *PDIA3*, *PDIA4*, and *P4HB.* Each clade contained one habu ortholog: habu1_s3443_g11710.t1 for the *P4HB* clade, habu1_s1390_g04983.t1 for the *PDIA3* clade, and habu1_s10239_g19902 for the *PDIA4* clade, indicating these habu gene model ID transcripts are the same genes as in humans. However, these molecular phylogenetic trees failed to support *P. flavoviridis* orthologs for *PDIA2* (Figure 3E) and *PDILT* (Figure 3D). Interestingly, genes for *PDILT* were not found in snakes.

These *PDI* gene family phylogenetic trees also contain clades other than *PDIA3, PDIA4, P4HB, PDIA2*, and *PDILT*, so we next examined molecular phylogenetic trees of the other *PDI* gene family members, including the human and habu genomes. Molecular phylogenetic analysis using ORTHOSCOPE showed that the habu genome contains one copy of each of the same genes as humans in the *PDIA5* (Supplementary Figure S1) and *PDIA6* clades (Supplementary Figure S2), since in each clade there is only one habu ortholog of the human gene, without any habu paralogs. These results indicate that in contrast to the extensive duplication of habu venom protein genes, genes for the PDI family are present as only single copies in the habu genome, although they are actively expressed in venom glands.

(b) Other PDI family-related members

The molecular phylogenetic tree of *ERP44* analyzed by ORTHOSCOPE using other *PDI* family member human *ERP44* (Supplementary Figure S5) as query included the four main clades (*ERP44*, *PDIA4*, *PDIA3*, and *P4HB*) that contained one habu ortholog each, habu1_s32648_g23040.t2 for the *ERP44* clade, habu1_s10239_g19902.t1 for the *PDIA4* clade, and habu1_s1390_g04983 for the *PDIA3* clade.

Molecular phylogenies of *AGR2* and *AGR3* using human genes as queries are shown in Supplementary Figures S6 and S7, respectively. First, the *AGR2* and *AGR3* trees both consisted of three main clades (*AGR3*, *AGR2*, and *TXNDC12*), and each clade contained one habu ortholog, habu1_s49803_g23446.t1 for the *AGR3* clade, habu1_s49803_g23445.t1 for the *AGR2* clade, and habu1_s2744_g09787.t1 for the *TXNDC12* clade.

Molecular phylogenies of *TMX1*, *TMX2*, *TMX3*, and *TMX4* (Supplementary Figures S8–S11, respectively) using human genes as queries indicated that a single habu ortholog exists in each clade: habu1_s1939_g07114.t1 for the *TMX1* clade, habu1_s5624_g16107.t1 for the *TMX2* clade, habu1_s4708_g14660.t1 for the *TMX3* clade, and habu1_s8507_g18763.t3 for the *TMX4* clade.

Molecular phylogenetic trees for *TXNDC5* and *TXNDC12* using each human gene as a query (Supplementary Figures S12 and S13) also indicated that the tree for *TXNDC5* contains three main clades (*PDIA4*, *PDIA6*, and *TXNDC5*), each of which includes a single habu ortholog: habu1s_10239_19902.t1 for the *PDIA4* clade, habu1_s604_g02858.t1 for the *PDIA6* clade, and habu1_s3682_g12384.t1 for the *TXNDC5* clade. The tree for *TXNDC12* contains the three main clades (*AGR2*, *AGR3*, and *TXNDC12*) (Supplementary Figure S13), and each clade contains a single ortholog: habu1_s49803_g23446.t1 for the *AGR3* clade, habu1_s49803_g23445.t1 for the *AGR2* clade, and habu1_s2744_g09787.t1 for the *TXNDC12* clade.

Molecular phylogenetic trees for *CASQ1* and *CASQ2* each using the human gene as a query showed that only a single habu ortholog exists in each: habu1_s2370_g08604.t2 for the *CASQ1* clade and habu1_s8721_g18867.t1 for the *CASQ2* clade (Supplementary Figures S14 and S15).

(c) Calreticulin members

In a similar fashion, to examine how many genes are present for *CALR* members in the habu genome, a molecular phylogenetic tree of *CALR* was constructed using ORTHOSCOPE, with human *CALR* as a query sequence (Figure 4). We found that a gene annotated as habu *CALR*, habu1_ s3471_g11839.t1, was included in the human *CALR* clade (Figure 4), indicating that the gene annotation for habu *CALR* is correct, since the habu gene annotated as *CALR* is an ortholog of human *CALR*. In addition, we found *CALR3, CANX* and *CLGN* homologs in the habu genome, habu1_ s2279_g08248.t1 for *CALR3* gene, habu1_s3471_g11839.t1 for *CALR* gene, habu1_ s4907_g14941.t1 for *CANX* gene (Supplementary Figure S17), habu1_ s2744_g09792.t1 for *CALRL* gene, and habu1_ s67827_g17445.t1 for *CLGN* gene (Figure 4). These observations were confirmed by molecular phylogenies of *CALR3* (Supplementary Figure S16), *CANX* (Supplementary Figure S17), and *CALRL* (Supplementary Figure S18). As with *PDI* and related families, members of the *CALR* family exist as single copies in the habu genome.

(d) Redox partner proteins of PDI family proteins, Ero1α and Ero1β

**Figure 5:**
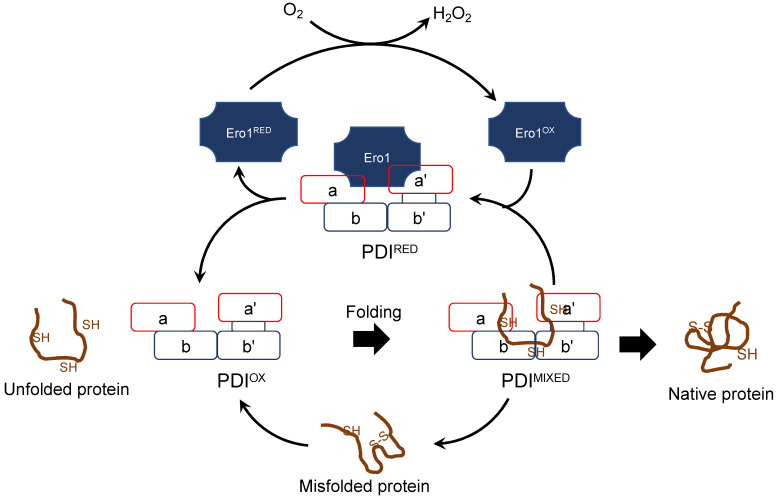
The relationship between PDI and Ero1 during protein folding by PDI. Redox regulation of PDI by Ero1 produces H_2_O_2_. PDI^OX^: oxidized PDI; PDI^RED^: reduced PDI; PDI^MIXED^: PDI with the bound substrate; Ero1*^OX^*: oxidized Ero1; Ero1*^RED^*: reduced Ero1. PDI domains are colored as shown in Figure 2. This figure was adapted and modified with permission from Ref. [22]. (2012, Claudio Hetz et al., John Wiley and Sons).

Ero1, which has two kinds of paralogs, Ero1α and Ero1β, is the re-oxidizing enzyme specific for P4HB in the *PDI* gene family (Figure 5) [18,19,20]. The *Ero1* gene is conserved evolutionarily from yeast to humans. Although the functional difference between Ero1α and Ero1β has not yet been clarified, it is well known that they show different expression profiles. *Ero1α* is expressed ubiquitously in various organs, while *Ero1β* is expressed only in secreting tissues [21]. We examined the number of *Ero1* copies in the habu genome and their expression profiles in different organs.

**Figure 6:**
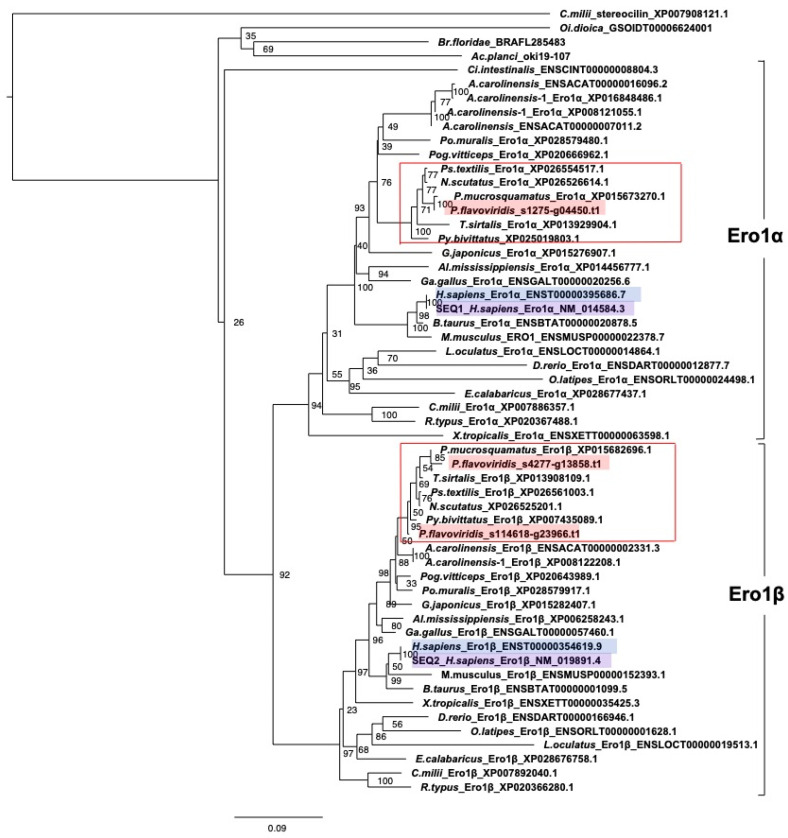
Molecular phylogenetic tree for *Ero1α* and *Ero1β*. This tree was estimated using ORTHOSCOPE with human *Ero1α* and *Ero1β* as query sequences (highlighted in purple). Sequences highlighted in blue indicate human orthologs. Those highlighted in pink represent habu (*P. flavoviridis*) orthologs, and those in red boxes represent other snake species. Values beside branches represent the percentages of times that nodes were supported in 100 bootstrap pseudoreplications implemented in ORTHOSCOPE. The scale bar indicates an evolutionary distance of 0.09 substitutions per position.

The molecular phylogeny of *Ero1* (Figure 6) indicated two distinct clades of *Ero1α* and *Ero1β*, each of which contains habu orthologs, habu1_s1275_g04450.t1 (*Ero1α*) and habu1_s4277_g13858.t1 (*Ero1β*). Although the habu ortholog of *Ero1β* was a single copy, the gene was divided into two scaffolds tentatively annotated as habu1_s114618_g23966 (N-terminal side) and habu1_s4277_g13858 (C-terminal side).

(e) TXN and PRX members

**Figure 7:**
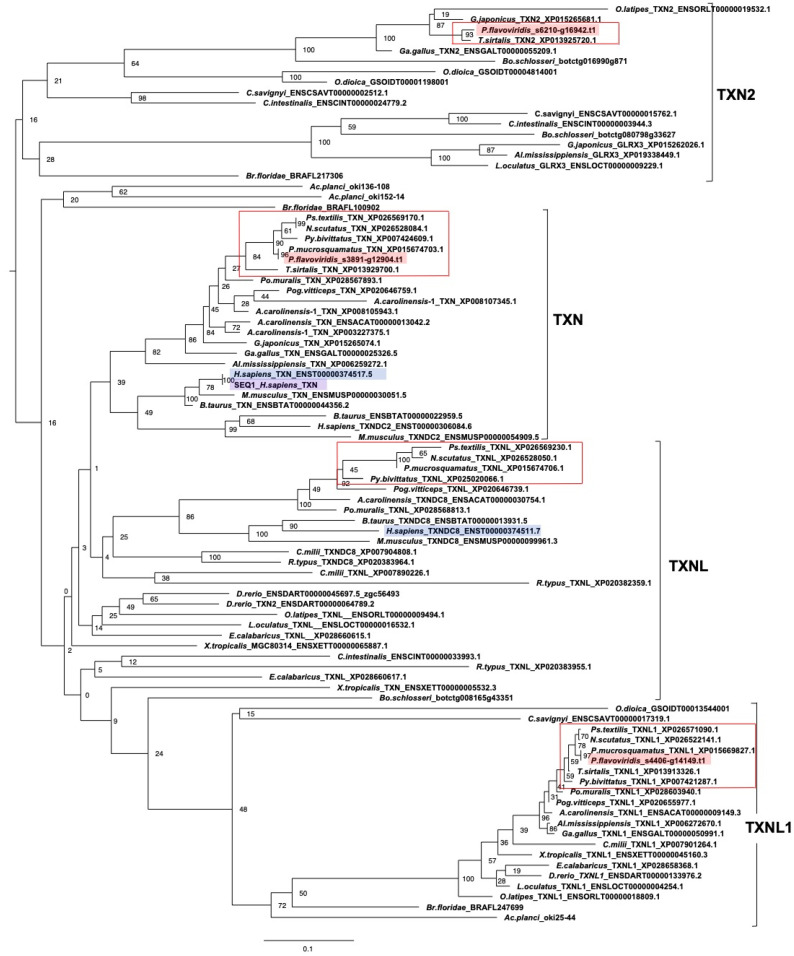
Molecular phylogenetic tree for TXN. This tree was estimated using ORTHOSCOPE with human *TXN* as the query sequence (highlighted in purple). The tree includes the clades *TXN2, TXN, TXNL*, and *TXNL1*. Sequences highlighted in blue indicate human orthologs. Those highlighted in pink represent habu (*P. flavoviridis*) orthologs, and those in red boxes represent other snake species. Values beside branches represent the percentages of times that nodes were supported in 100 bootstrap pseudoreplications implemented in ORTHOSCOPE. The scale bar indicates an evolutionary distance of 0.1 substitutions per position.

The molecular phylogeny of thioredoxin (*TXN*) members using ORTHOSCOPE, with human *TXN* as query sequences is shown in Figure 7. The tree consists of four main clades, namely *TXN2*, *TXN*, *TXNL*, and *TXNL1*, and clades *TXN2*, *TXN*, and *TXNL1* each contained one habu ortholog: habu1_s6210_g16942.t1 for *TXN2* clade, habu1_s3891_g12904.t1 for *TXN* clade, and habu1_s4406_g14149 for *TXNL1* clade. However, we failed to find an ortholog for *TXNL*.

**Figure 8:**
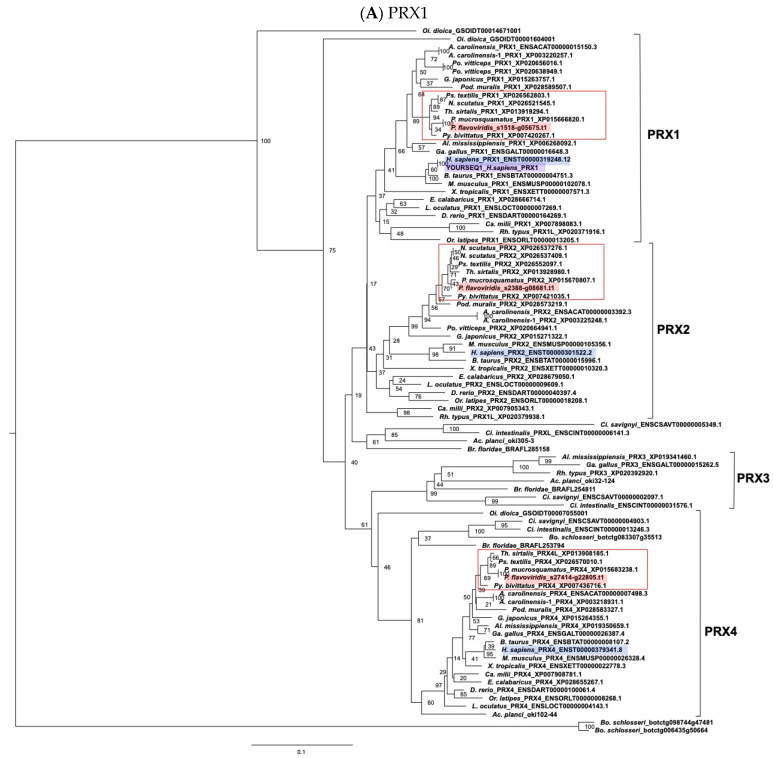

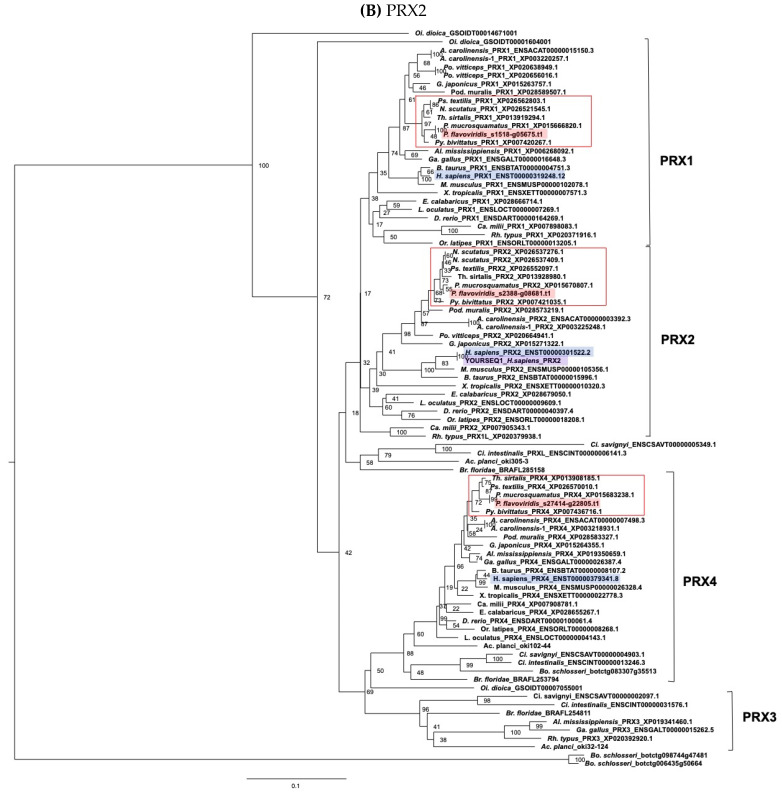

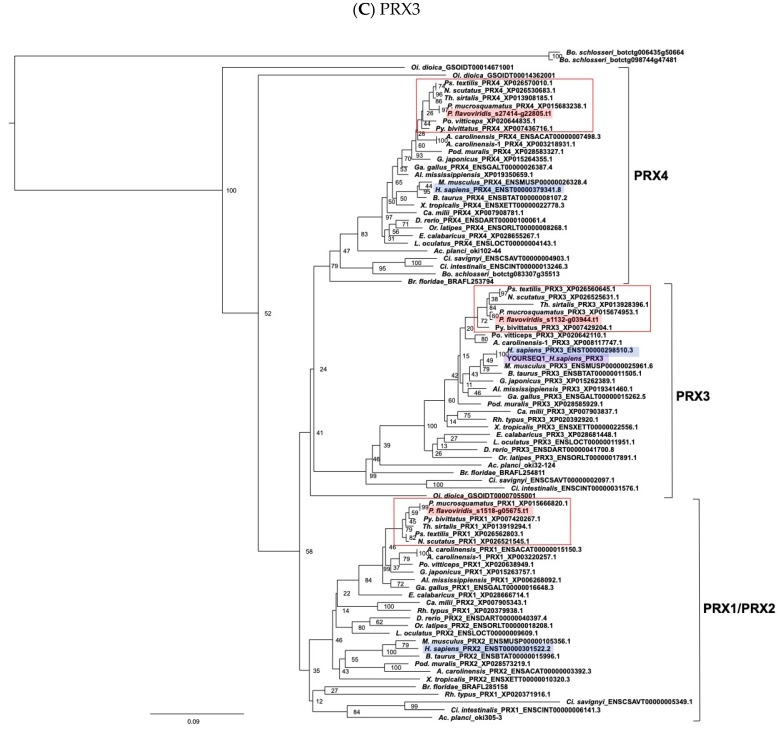

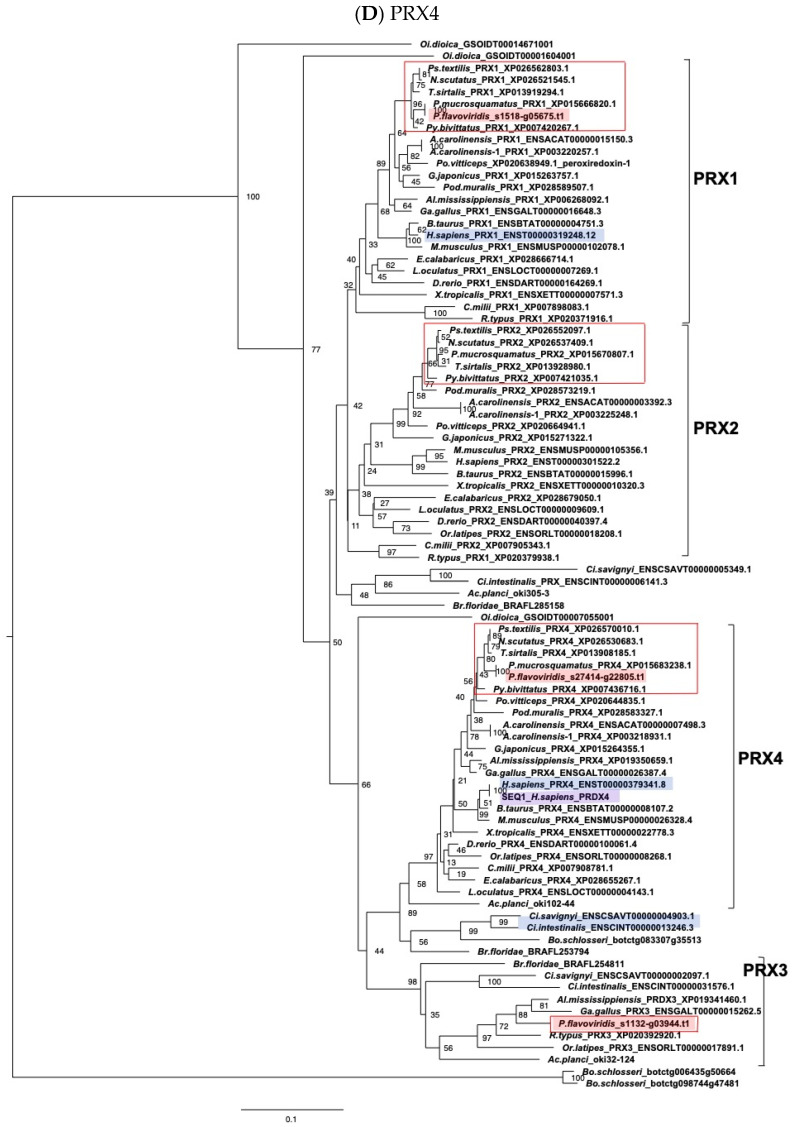
Molecular phylogenetic tree for *PRX1, PRX2, PRX3*, and *PRX4.* These trees were estimated using ORTHOSCOPE with human *PRX1* (**A**), *PRX2* (**B**), *PRX3* (**C**), and *PRX4* (**D**) as query sequences (highlighted in gray-colored), respectively. Sequences highlighted in blue indicate human orthologs. Those highlighted in pink represent habu (*P. flavoviridis*) orthologs, and those in red boxes represent other snake species. Values beside branches represent the percentages of times that nodes were supported in 100 bootstrap pseudoreplications implemented in ORTHOSCOPE. The scale bar indicates an evolutionary distance of 0.1 substitutions per position.

Molecular phylogenic trees of peroxiredoxin (*PRX*) isoforms genes were analyzed, with human *PRX1* (Figure 8A), *PRX2* (Figure 8B), *PRX3* (Figure 8C) and *PRX4* (Figure 8D) as query sequences. The tree comprises four discrete clades of *PRX1, PRX2, PRX3*, and *PRX4*. Each *PRX* clade contained a habu ortholog, habu1_s1518_g05675.t1 for the *PRX1* clade, habu1_s2388_g08681.t1 for the *PRX2* clade, habu1_s1132_g03944.t1 for the *PRX3* clade, and habu1_s27414_g22805.t1 for the *PRX4* clade.

### 2.4. Characterization of the Habu SELENOM Gene

**Figure 9:**
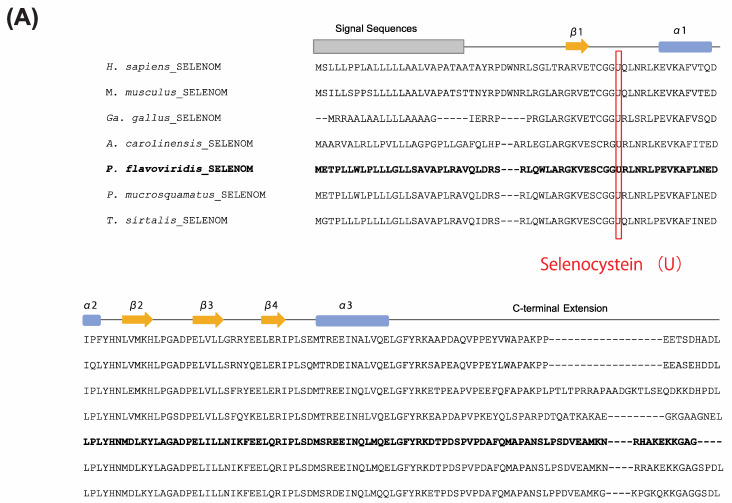

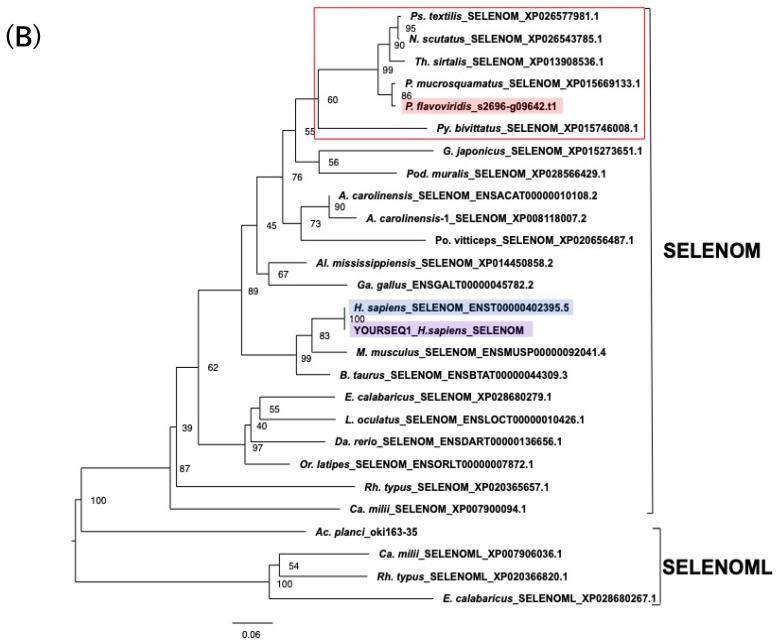
Aligned amino acid sequences (**A**) and a molecular phylogenetic tree (**B**) for *SELENOM*. The conserved selenocysteine (U) residue, which is characteristic of selenoproteins, is shown in red. Secondary structures, α-helix and β-strand, are shown with blue boxes and orange arrows, respectively. The signal sequence is also shown in gray. This tree was estimated using ORTHOSCOPE with human *SELENOM* as the query sequence (highlighted in purple). Sequences highlighted in blue indicate human orthologs. Those highlighted in pink represent habu (*P. flavoviridis*) orthologs, and those in red boxes represent other snake species. Values beside branches represent the percentages of times that nodes were supported in 100 bootstrap pseudoreplications implemented in ORTHOSCOPE. The scale bar indicates an evolutionary distance of 0.06 substitutions per position.

Although the *PDI* gene family has been studied in detail, *SELENOM* has been poorly characterized, including its exact function. Selenocysteine (U) is a unique, rare amino acid encoded by the codon, UGA. It is highly conserved in selenoproteins. We therefore performed an alignment with habu Selenoprotein M and confirmed that the position of selenocystein (U) is highly conserved in all gene sequences, including habu Selenoprotein M (Figure 9A).

In addition, to confirm whether the habu gene annotated as *SELENOM*, habu1_s2696_g09642.t1, is a human *SELENOM* ortholog, a molecular phylogenetic tree of *SELENOM* was constructed using ORTHOSCOPE, with human *SELENOM* as the query sequence (Figure 9B). The resulting phylogenetic tree includes a single habu gene annotated as *SELENOM* in the same clade as human *SELENOM*, and we found no paralogs of habu *SELENOM*.

### 2.5. Expression Profiles of Genes Encoding PDI and PDI-Related Proteins in the Habu

To determine the functions of candidate enzymes or chaperones of venom protein modification more specifically, we next investigated expression profiles of not only PDI family genes, but also their redox partner proteins in various organs and tissues of *P. flavoviridis*, including venom glands (Table 2). First, we focused on organ-specific genes in venom glands. Four genes for venom protein folding candidate enzymes or chaperones, *P4HB*, *SELENOM*, *CALR*, and *PDIA3*, showed higher expression levels in venom glands (TPM > 1000) than in other organs. However, except for *SELENOM*, they all showed high and ubiquitous expression in all organs (Table 2).

For example, *P4HB* showed a considerably higher expression level in venom glands (TPM (venom gland1/venom gland2) = 8572.4/13,303.9) than in other organs, in the range of (TPM =) 213–4084 (40 times higher than brain). *CALR* and *PDIA3* also showed high expression levels in venom glands (361.1/1422 and 2266.3/1085.9, respectively) and in other organs. *SELENOM* showed high expression levels in venom glands (1514.7/1928.2), and in other organs, except for the liver, ovary, kidney, stomach, and small intestine. In addition to these genes of the four protein-modification enzyme candidates, *PDIA4*, *PDIA6 (P5)*, *TXNDC12*, and *CASQ1*, also showed high expression levels in venom glands (95.7/172.3, 181.2/388.5, 99.8/160, and 3732.7/97.1, respectively), while the expression levels of other protein-modification enzymes genes, such as *PDIA2*, *PDIA5*, *ERp46*, *ERp27*, *ERp29*, *TMX1*, *TMX2*, *TMX3*, *TMX4*, *AGR2*, *AGR3*, *DNAJC10*, and *CASQ2*, in the venom gland were lower than those in other organs.

Among the redox partner proteins of PDIs, PRX4 is an ER-localized PRX isoform that reduces hydrogen peroxide produced during Ero1-mediated oxidation of P4HB [23,24,25]. PRX4 oxidizes PDIA6 and TXNDC5 most efficiently [19,24,26]. In habus, *PDIA6* tends to have ubiquitously high expression, and especially in venom glands and fetal fibroblasts, specifically (TPM = 388.5 and 541.2, respectively). Additionally, in habus, all organs, including venom glands, showed high expression of *PRX4* (TPM = 229.4).

On the other hand, the expression level of *Ero1* in various habu organs showed that two kinds of paralogs of *Ero1*, *Ero1α*, and *Ero1β*, exhibit different expression profiles. *Ero1α* was ubiquitously expressed in all organs, but at low levels (TPM = 0.3–29.3). The expression level of *Ero1α* was especially low (TPM = 3.3) in venom glands, whereas *Ero1β* was comparatively highly expressed in venom glands (TPM = 75.1 to 247.6) as well as in the ovary, spleen, pancreas, and nose (TPM = 182.8–498.9). Thus, the expression levels of *Ero1α* and *Ero1β* differ, depending on organs and tissues, as noted in other vertebrates [27].

### 2.6. No Accelerated Evolution of Genes for Candidate Venom Protein Modification Enzymes or Chaperones

Our previous study of the habu genome revealed that most venom protein genes have been extensively duplicated and that major venom genes including *svMPs* exhibit accelerated evolution when examined by *K_A_/K_S_* analysis. In contrast, as shown here, genes involved in the modification of venom proteins are present as single copies in the habu genome. However, since these genes are also highly expressed in the venom gland, we examined whether accelerated evolution occurred in these genes by calculating *K_A_/K_S_* values using the Nei–Gojobori method.

We first examined the possible accelerated evolution of *P4HB* and *PDIA3*. However, *K_A_/K_S_* analysis against *P4HB* and *PDIA3* genes showed no accelerated evolution of the *P4HB* and *PDIA3* genes (Supplementary Figure S19). Further, *K_A_/K_S_* analysis indicated that habu *CALR* and *SELENOM* also showed no accelerated evolution (Supplementary Figure S20). *K_A_/K_S_* values for *PDI* family genes, *P4HB* and *PDIA3*, were almost 0, indicating that these two genes are highly conserved.

## 3. Discussion

In this study, in addition to genes that encode venom and ribosomal proteins, genes that encode PDI family proteins (human *P4HB* and *PDIA3* orthologs), Selenoprotein M (*SELENOM)*, and Calreticulin (*CALR)*, are highly expressed in habu venom glands. Since these enzymes or chaperones are involved in protein modification and folding in the cellular secretory pathway, the present results suggest the most likely involvement of PDI, PDIA3, Selenoprotein M, and Calreticulin in protein folding and modification during snake venom production as candidate modification enzymes or chaperones. Therefore, it is likely that PDI, PDIA3, Selenoprotein M, and Calreticulin are critical to confer toxic functions upon the disulfide bond-rich venom transcriptome.

Although studies of venom transcriptomes or proteomes have been conducted for many venomous snakes [3,4,5], there have been only a few reports focused on non-toxin transcripts or proteins, especially genes for enzymes that are involved in venom protein modification. In transcriptomic analysis of venom glands of the eastern diamondback rattlesnake (*Crotalus adamanteus*), Rokyta et al. (2012) [14] demonstrated that genes for PDIs are highly expressed in venom glands. Furthermore, proteomic analysis of *Bothrops jararaca* venom glands [15] found that proteins from the endoplasmic reticulum (ER), such as PDI and GPR78, and cytoplasmic proteins, such as 40S ribosomal protein, are enriched in venom glands. Our present transcriptome analysis indicated that *P4HB, PDIA3, SELENOM*, and *CALR*, which are non-toxin genes, are among the 50 most highly expressed venom gland genes. Consistently, the 20 most highly expressed non-toxin transcripts listed by Rokyta et al. (2012) [14] included PDI, calreticulin, and PDIA3. Thus, our findings for habu venom glands support results for other pit vipers, which manifest compositional similarities.

In this context, with transcriptomic data for the Indian cobra (*Naja naja*), venom of which is rich in post-synaptic neurotoxins, Suryamohan et al. (2020) [28] showed the high expression of *PDI* (log (CPM) = 13.27~15.65 (*n* = 6)), as well as the moderately high expression of *PDIA3* [28]. While their transcriptomic data for 109 differentially upregulated genes (DUGs) in cobra venom glands did not include *P4HB* and *PDIA3*, these genes were more highly expressed in venom glands than in other organs. This seems reasonable since *PDIs* are expressed ubiquitously due to their essential functions as protein modification enzymes or chaperones. DUG analysis is undoubtedly useful to find genes of venom proteins since venom proteins are exclusively expressed in venom glands. However, this type of analysis may not always be useful to find genes that are ubiquitously and highly expressed, but more so in specific organs. When focusing on essential genes encoding non-toxic proteins, we need to consider the most appropriate analytical method. Nevertheless, all data shown herein for various snake species allow us to speculate that PDIs are essential for the modification and folding of venom proteins with abundant cysteines and multiple disulfide bonds.

Given that PDI works together with Ero1 as the primary catalyst for disulfide bond formation (Figure 5), we surmised that high expression of habu *PDI* accompanies high expression of habu *Ero1*. Indeed, our transcriptomic data from habu venom glands indicate high co-expression of *Ero1β* and *PDI*, whereas *Ero1α* is expressed to a much lesser extent. This was an unexpected result. However, previous studies demonstrated that *Ero1β* is constitutively and abundantly expressed in professional secretory tissues and can be strikingly induced in the course of the unfolded protein response [27,29]. Moreover, recombinant human Ero1β is twice as active as Ero1α in enzymatic assays [30]. Thus, it is possible that PDI is oxidized by Ero1β more efficiently than by Ero1α, leading to the efficient catalysis of oxidative protein folding by the PDI-Ero1β combination [30]. The present finding of the high expression of *PDI* together with *Ero1β* supports our notion that habu PDI could be the primary venom protein modification enzyme.

In this study, ORTHOSCOPE, the leading tool for phylogenetic analysis, enabled us to estimate phylogeny for all *PDI* family orthologs and paralogs and their redox partner genes, *SELENOM* and *CALR.* This program is a species tree-based ortholog or paralog relationship identification tool, and all orthologs and paralogs for a specific gene are estimated automatically using this software [16]. Our previous data showed that each habu venom protein gene has many paralogs and undergoes accelerated evolution. In contrast, habu genes encoding candidate venom protein modification enzymes are orthologs for those of humans, and consistently there are no paralogs. Furthermore, our *K_A_/K_S_* analysis indicates that these genes never undergo accelerated evolution. In contrast to habus, proteomic analysis of cone snails, which have conotoxin, by Safavi-Hemami et al. (2011) demonstrated multiple PDI isoforms in cone snail venom glands, suggesting that cone snail *PDI* does undergo accelerated evolution [31]. Therefore, this contrast suggests that in habus, venom modification enzymes modify a wide variety of venom proteins, without requiring multiple isoforms derived from gene duplication or accelerated evolution. This difference between habus and cone snails may also help clarify molecular mechanisms for venom protein modification.

Variables relative to venom gland sampling conditions can also affect results. Luna et al. (2013) [15] indicated that a venom production cycle has two stages: a quiescent stage and an active stage. In *Bothrops jararaca*, this cycle continues for about 30–50 days [15]. Moreover, they demonstrated that the kinds and quantities of proteins detected in these two steps were totally different, suggesting that although there are differences in the length of this cycle among snake species, timing is quite important for venom gland sampling for transcriptomic or proteomic analysis. Results of venom gland proteomic analysis change dramatically depending on the timing of sampling [15]. Thus, in this study, the number of samples was insufficient for statistical analysis. However, our results are consistent with those of other studies. Time-course single-cell RNA-seq analysis might allow for a better understanding of the accurate transition of the venom glands expression profile according to the entire timing of the cycle, as well as mechanisms of venom protein modification.

## 4. Conclusions

In this study, we identified four non-toxin genes quite highly expressed in venom glands of *P. flavoviridis*, namely *P4HB, PDIA3, SELENOM*, and *CALR*, and in general all of which encode protein folding enzymes or molecular chaperones in the ER. These are single-copy genes and are highly conserved among *Protobothrops* species, in contrast to the multiple copies and accelerated evolution of venom protein genes. Gene for a redox partner protein of PDI, *Ero1β* is also highly expressed in venom glands. The highly active expression of *P4HB, PDIA3, SELENOM*, and *CALR* in habu venom glands, which is also supported by previous reports, together with the disulfide bond-rich nature of venom proteins and the high co-expression of PDIs’ redox partner genes, suggests that PDI, PDIA3, Selenoprotein M, and Calreticulin are strongly associated with venom protein modification. It is most likely that folding enzymes or molecular chaperones such as habu PDIs are critical to endow them with toxic functions.

## 5. Materials and Methods

### 5.1. Transcriptomic Data and Comparative Analyses

For transcriptomic analyses, a total of 22 samples were used as follows: 20 samples for 18 tissues and organs from two adult animals, and 2 samples for 2 tissues and organs from one neonate, including two venom glands specimens from two adult (sample name: venom gland-specimen#1, venom gland-specimen#2); two brains specimens from two adult (brain-specimen#1, brain-specimen#2); one specimen of eye, nose, infrared sensing pit organ, and venom fang-forming tissue, from one female adult; one specimen of lungs, liver, kidneys, small intestine, colon, stomach, pancreas, heart, masseter muscle, spleen, ovary, and accessory glands from the other female adult; one neonate venom gland tissue, as well as fibroblast cells, from one neonate. We extracted total RNA using a standard TRIzol protocol (Thermo Fisher Scientific, Waltham, MA, USA), and prepared cDNA libraries using an NEBNext^®^ Ultra™ Directional RNA Library Prep Kit for Illumina (New England Biolabs, Ipswich, MA, USA). RNA quality was checked with an Agilent Technologies 2100 Bioanalyzer using an Agilent RNA 6000 Nano Kit. Sequencing was performed using an Illumina Hiseq2500. De novo assembly of whole RNA sequence reads was performed using a de Bruijn graph-based program, Trinity [32,33]. Assembled transcripts were annotated with BLASTX against UniProt. All Illumina reads are available from DRA under accession No. DRA006600.

Raw Illumina reads were quality filtered and trimmed with Trimmomatic (v0.36) [34]. After adapter trimming and quality filtering of RNA-seq reads, index files for Kallisto [35] psudoalignment were generated with transcriptome information of our habu gene model. Gene expression levels (TPM) were calculated with Kallisto.

The 50 most highly expressed genes in venom glands were identified, and high-expression profiles of genes in the venom gland are listed in order (Table 1 and Supplementary Table S1).

### 5.2. Molecular Phylogenetic Analysis

We performed molecular phylogenetic analysis using ORTHOSCOPE, which is a novel pipeline for molecular phylogenetic analysis [16]. This unique pipeline, which is a species tree-based ortholog identification tool, automatically estimates the phylogenetic trees, which can detect all orthologs and paralogs automatically from whole genomic databases of selected species among all registered species. The software judges the range of the ortholog clade, which is the monophyletic group (clade), including the operational taxonomic unit (OTU) of interest, based on the topology of the species trees, which are given as quide trees. All phylogenetic trees were rearranged with Fig-TREE using the Newick format results of ORTHOSCOPE analysis.

To identify orthologs or paralogs of not only *P4HB*, *PDIA3*, *SELENOM*, and *CALR*, but also the other *PDI* family genes and *PDI* related redox partner genes, phylogenetic analysis was conducted with protein-coding DNA sequences of human *PDIA3*, *PDIA4*, *P4HB*, *PDILT*, *PDIA2*, *CALR*, both of *Ero1α* and *Ero1β*, *TXN*, *PRX1*, *PRX2*, *PRX3*, *PRX4*, *SELENOM*, *PDIA5*, *PDIA6*, *ERP27*, *ERP29*, *ERP44*, *AGR2*, *AGR3*, *TMX1*, *TMX2*, *TMX3*, *TMX4*, *TXNDC5*, *TXNDC12*, *CASQ1*, *CASQ2*, *CALR3*, *CANX*, and *Danio rerio CALRL/CALR3b* as queries, respectively, using ORTHOSCOPE, a species tree-based ortholog identification tool [16] with the following settings: analysis group, all Lepidosauria species and the default setting species for vertebrata analysis; E-value threshold for reported sequences, 1 × 10^−3^; number of hits to report per genome, 3; aligned site rate threshold within unambiguously aligned sites, 0; data set, DNA (Exclude 3rd); rearrangement BS (bootstrap) value threshold, 60%. In ORTHOSCOPE analysis, to construct neighbor joining (NJ) trees of all the above genes, all of the following analysis were carried out: amino acid sequences of each query gene were aligned with MAFFT (v7.221) [36] using the –auto strategy. Unaligned regions were trimmed with TrimAl (v1.2rev59) [37] using the –gappyout option. To generate nucleotide alignments, the corresponding DNA sequences were converted into the amino acid alignment using PAL2NAL [38]. For phylogenetic analyses, the NJ method [39] implemented in APE in R [40] for DNA alignments and FastME [41] for amino acid alignments were applied. Analyses of DNA alignments employ the most parameter-rich model in the program, the TN 93 model [42], with a gamma-distributed rate for site heterogeneity [43]. Analyses of amino acid alignments employ a widely used substitution model for nuclear gene analysis, the WAG model [44], with the gamma model. To evaluate the robustness of internal branches, 100 bootstrap replications are calculated for each data set.

### 5.3. Alignment and Molecular Evolutionary Analysis

We conducted the alignment of amino acid sequences of Selenoprotein M in a representative vertebrate using MUSCLE (v.3.8.31) alignment algorithms [45]. For *K_A_/K_S_* analysis, DNA sequences were aligned by using the ClustalW multiple alignment program (http://clustalw.ddbj.nig.ac.jp (accessed on 31 August 2021)) [46]. Due to the complicated structure of the genes for this *K_A_/K_S_* analysis, all alignments were manually curated. For pairwise comparisons of nucleotide sequences of each the organism genes, the number of nucleotide substitutions per synonymous site (*K*_S_) and per non-synonymous site (*K*_A_) for protein-coding regions was computed according to the Nei–Gojobori method [47] using the Sqdif Plot online (http://www.gen-info.osaka-u.ac.jp/~uhmin/study/sqdifPlot/index.html (accessed on 31 August 2021)).

## Figures and Tables

**Table 1:** Highly expressed genes in venom glands from an adult habu (venom gland-specimen#2: a main adult specimen).

Gene Model ID	Gene Name	Annotation by Blast2GO	TPM	
habu1_s258676_g24318.t1	*CNP01*	Bradykinin-potentiating and C-type natriuretic peptides	438,781.0	Venom protein
habu1_s47459_g23397.t1	*svPLA2-02* , *svPLA2-03*	phospholipase a2	88,799.1	Venom protein
habu1_s14911_g21429.t1	*svMP06*	p-ii partial	76,130.9	Venom protein
habu1_s9571_g19434.t1	*svPLA2-01*	phospholipase a2	44,208.4	Venom protein
habu1_s9570_g19432.t1	*svPLA2-07*	phospholipase a2	34,366.9	Venom protein
habu1_s9571_g19433.t1	*svPLA2-08*	phospholipase a2	31,067.9	Venom protein
habu1_s14911_g21430.t1	*svMP07* , *svMP08*	snake venom metalloproteinase	30,773.7	Venom protein
habu1_s10061_g19809.t1	*svCTLP06*	flavocetin-A beta	15,201.6	Venom protein
habu1_s3443_g11710.t1	*P4HB*	protein disulfide-isomerase	8572.4	Modification enzyme for proteins analyzed in this study
habu1_s10061_g19810.t1	*svCTLP03* , *svCTLP04*, *svCTLP07*	snaclec stejaggregin-a subunit alpha	6786.0	Venom protein
habu1_s3258_g11210.t1	*svMP04*	metalloprotease piia	4821.0	Venom protein
habu1_s3168_g10977.t1	*svCTLP08*	venom c-type lectin mannose binding isoform 1	3305.5	Venom protein
habu1_s3258_g11211.t1	*svMP05*	snake venom metalloproteinase	2659.2	Venom protein
habu1_s6789_g17480.t1	*svSP04*	snake venom serine protease 2a homolog	2416.4	Venom protein
habu1_s2955_g10552.t1		elongation factor 1-alpha 1	2283.4	
habu1_s402940_g24950.t1	*LAAO01*	l-amino acid oxidase	2124.7	Venom protein
habu1_s158_g00895.t1		forkhead box protein j1	2102.3	
habu1_s5309_g15576.t1		40S ribosomal protein S25	1678.5	Ribosomal protein
habu1_s2696_g09642.t1	*SELENOM*	Selenoprotein M	1514.7	Modification enzyme for proteins analyzed in this study
habu1_s68614_g23647.t1		60s ribosomal protein l7	1508.8	Ribosomal protein
habu1_s1917_g07050.t1		60s ribosomal protein l7a	1458.7	Ribosomal protein
habu1_s4106_g13431.t1		serine partial	1457.8	Venom protein
habu1_s3471_g11839.t1	*CALR*	Calreticulin	1422.0	Modification enzyme for proteins analyzed in this study
habu1_s4158_g13542.t1		55 kda erythrocyte membrane protein	1406.7	
habu1_s4037_g13262.t1		low quality protein: glutathione peroxidase 3	1337.9	
habu1_s3765_g12603.t1		40s ribosomal protein s3a	1259.4	Ribosomal protein
habu1_s22025_g22470.t1	*CRISP01* , *CRISP02*	cysteine-rich venom partial	1201.7	Venom protein
habu1_s22023_g22469.t1	*svSP11*	snake venom serine protease 2	1194.1	Venom protein
habu1_s6274_g17028.t1		ubiquitin-40s ribosomal protein s27a	1159.9	Ribosomal protein
habu1_s1466_g05403.t1		peptidyl-prolyl cis-trans isomerase a-like	1149.4	
habu1_s399953_g24864.t1	*svMP09* , *svMP10*	metalloproteinase precursor	1138.2	Venom protein
habu1_s1390_g04983.t1	*PDIA3*	protein disulfide-isomerase a3	1085.9	Modification enzyme for proteins analyzed in this study
habu1_s2087_g07635.t1		ribosomal protein	1075.8	Ribosomal protein
habu1_s7284_g17906.t1		40s ribosomal protein s13	1019.6	Ribosomal protein
habu1_s9530_g19385.t1		Elongation factor 1-beta	999.5	
habu1_s4833_g14854.t1		tmsb4x partial	971.8	
habu1_s2449_g08987.t1		60s ribosomal protein l31	965.9	Ribosomal protein
habu1_s7991_g18513.t1		40s ribosomal protein partial	934.3	Ribosomal protein
habu1_s37466_g23178.t1		growth-related translationally controlled tumor protein	928.1	
habu1_s16120_g21649.t1		60s ribosomal protein l18a	906.8	Ribosomal protein
habu1_s7978_g18472.t1		60s acidic ribosomal protein p2	902.7	Ribosomal protein
habu1_s2862_g10314.t1	*svMP01* , *svMP02*, *svMP03*, *svMP11*, *nvMP57*	disintegrin and metalloproteinase domain-containing protein 28	902.6	Venom protein
habu1_s1408_g05043.t1		60s ribosomal protein l5	892.3	Ribosomal protein
habu1_s74_g00428.t1		60s ribosomal protein l11	872.1	Ribosomal protein
habu1_s6028_g16570.t1	*sv5Nase*	ecto-5 -nucleotidase	852.8	Venom protein
habu1_s399842_g24584.t1		60s ribosomal protein l6	852.5	Ribosomal protein
habu1_s188_g01057.t1		60s ribosomal protein partial	852.1	Ribosomal protein
habu1_s1608_g06024.t1		40s ribosomal protein s4	837.9	Ribosomal protein
habu1_s1167_g04045.t1		polyadenylate-binding protein 1 isoform x1	834.0	
habu1_s2592_g09366.t1		cytoplasmic 2	819.2	

Transcripts are expressed as “Transcripts per million” (TPM). red: Venom protein genes, blue: Protein modification genes analyzed in this study.

**Table 2:** Gene expression level of *P4HB*, *PDIA3*, *SELENOM*, *CALR*, *PDI* family genes and *PDI* family related genes by RNA-seq analysis of tissues and organs of *Protobothrops flavoviridis*.

Gene Model ID	Gene Name	Venom Gland (Specimen #1)	Venom Gland (Specimen #2)	Venom Fang Forming Tissue	Lung	Liver	Kidney	Small Intestine	Colon	Stomach	Pancreas	Heart	Masseter Muscle	Brain (Specimen #1)	Brain (Specimen #2)	Eye	Nose	Pit, Infrared Sensing Organ	Spleen	Ovary	Fetal Fibroblast
habu1_s2696_g09642	*SELENOM*	1928.2	1514.7	273.2	104.5	3.9	12.1	38.7	179.5	31.5	311.1	53.2	15.0	178.8	72.2	675.8	1184.5	407.1	213.1	4.1	261.9
habu1_s3471_g11839	*CALR*	361.1	1422.0	531.0	258.3	71.7	172.5	295.9	375.4	167.4	78.4	139.7	141.8	281.4	319.0	184.1	569.8	413.7	159.2	464.0	1859.1
habu1_s3443_g11710	*P4HB*	13,303.9	8572.4	955.2	1564.9	3765.2	1874.4	4083.9	1789.5	2381.0	2265.3	349.7	601.7	249.8	213.4	771.6	1268.3	1256.5	1916.5	645.9	917.0
habu1_s2789_g10033	*PDIA2*	0.1	0.2	0.4	0.7	0.1	0.0	0.4	0.3	59.1	0.1	0.2	0.6	0.6	0.5	0.3	1.1	0.5	0.1	0.5	0.2
habu1_s1390_g04983	*PDIA3*	2266.3	1085.9	601.2	856.4	420.2	614.1	1074.1	857.4	675.8	425.2	272.4	214.7	270.0	276.4	434.1	892.6	728.6	455.6	427.0	872.6
habu1_s10239_g19902	*PDIA4*	95.7	172.3	159.4	164.9	68.8	83.5	61.6	121.6	164.6	129.3	63.4	29.2	47.0	50.3	82.5	141.3	182.9	122.6	134.7	252.0
habu1_s2439_g08885	*PDIA5*	15.8	27.7	35.9	14.1	9.9	7.1	26.8	44.1	24.9	3.0	5.6	1.5	17.4	13.8	14.4	38.1	34.2	6.5	72.8	79.6
habu1_s604_g02858	*PDIA6*	181.2	388.5	146.1	71.4	18.6	33.8	35.9	146.4	40.3	29.6	34.4	30.5	68.4	66.3	62.9	195.6	149.5	83.5	135.3	541.2
habu1_s25599_g22719	*ERp27*	0.1	0.7	1.5	2.9	0.0	1.2	0.2	2.4	0.7	402.7	0.5	1.6	1.1	0.8	1.6	0.6	4.9	240.8	0.3	2.0
habu1_s66_g00353	*ERp27*	15.7	5.9	119.4	203.5	20.6	32.1	41.4	170.0	50.9	885.9	49.8	12.0	275.5	312.6	39.9	86.8	134.5	791.5	6.4	262.6
habu1_s399842_g24580	*ERp29*	53.1	38.4	112.9	84.2	19.5	22.6	100.1	109.9	43.6	10.1	36.5	15.4	44.9	48.3	40.5	180.2	102.4	32.8	52.0	239.2
habu1_s32648_g23040	*ERp44*	15.2	15.5	45.5	17.9	5.8	13.1	19.6	22.8	15.6	4.3	12.3	4.5	24.3	22.9	14.8	32.1	27.2	10.4	42.5	25.7
habu1_s1939_g07114	*TMX1*	6.9	5.0	23.1	25.4	17.1	22.9	20.3	23.8	11.2	4.7	21.4	11.9	30.5	38.9	12.9	21.1	21.6	17.8	46.5	26.3
habu1_s5624_g16107	*TMX2*	18.9	9.5	36.8	43.6	25.9	45.5	30.2	35.9	42.8	9.1	93.2	29.3	122.0	142.7	35.3	43.4	42.5	14.8	101.2	26.9
habu1_s4708_g14660	*TMX3*	8.0	3.8	43.3	23.1	5.4	19.7	13.0	19.3	13.6	2.2	20.5	4.5	42.7	43.6	15.3	54.6	33.7	12.5	25.6	56.0
habu1_s8507_g18763	*TMX4*	1.7	1.4	9.9	8.6	25.7	14.9	6.0	6.6	3.1	0.5	3.4	1.0	19.5	16.5	6.5	5.5	9.5	4.5	51.4	10.8
habu1_s3682_g12384	*TXNDC5*	53.1	38.4	112.9	84.2	19.5	22.6	100.1	109.9	43.6	10.1	36.5	15.4	44.9	48.3	40.5	180.2	102.4	32.8	52.0	239.2
habu1_s2744_g09787	*TXNDC12*	99.8	160.0	134.1	101.1	28.8	51.5	72.6	75.0	58.3	21.8	54.4	24.5	99.7	70.7	60.8	127.3	127.1	50.3	49.0	224.3
habu1_s49803_g23445	*AGR2*	22.4	3.0	406.3	183.2	0.3	77.9	199.0	777.2	649.1	1.8	0.5	12.3	6.8	1.4	33.8	884.0	356.9	0.7	0.7	0.2
habu1_s49803_g23446	*AGR3*	0.0	0.0	0.3	0.0	0.6	0.1	0.0	0.0	0.1	0.5	0.2	0.1	0.0	0.1	0.4	11.4	0.6	0.1	0.3	0.0
habu1_s2168_g07811	*DNAJC10*	2.2	5.7	10.8	3.3	0.6	4.8	4.5	6.8	9.6	0.4	11.0	5.2	18.1	16.3	3.8	10.0	10.7	2.9	102.0	58.3
habu1_s2370_g08604	*CASQ1*	3732.7	97.1	485.9	6.5	4.7	12.9	118.6	5.1	16.0	1.2	19.3	42,708.5	61.8	59.8	142.0	114.3	1277.6	4.3	39.8	9.4
habu1_s8721_g18867	*CASQ2*	1.3	0.1	0.5	2.6	0.0	0.5	0.2	1.9	0.8	0.1	662.8	11.7	22.2	20.0	22.2	0.9	26.3	1.3	1.7	0.6
habu1_s1275_g04450	*Ero1α*	1.8	3.3	21.0	9.2	1.2	3.2	7.4	13.3	16.7	0.3	2.0	0.4	25.2	18.9	4.6	12.0	5.9	3.3	7.7	29.3
habu1_s114618_g23966 habu1_s4277_g13858	*Ero1β*	75.1	247.6	91.8	44.0	50.4	24.2	20.7	37.1	45.1	182.8	21.5	8.5	49.6	59.8	60.1	498.9	125.2	200.8	255.9	117.9
habu1_s27414_g22805	*PRX4*	211.6	229.4	118.4	280.8	342.2	98.1	245.3	154.7	157.9	229.6	71.1	28.8	52.8	53.5	68.6	121.5	144.5	187.7	104.7	215.9

## Data Availability

Not applicable.

## Supplementary Materials

The following are available online at https://www.mdpi.com/article/10.3390/toxins14050300/s1, Figure S1: Molecular phylogenetic tree estimated using ORTHOSCOPE with human *PDIA5* as the query sequence (highlighted in purple). Sequences highlighted in blue indicate human orthologs. Those highlighted in pink represent habu (*P. flavoviridis*) orthologs, and those in red boxes represent other snake species. Values beside branches represent percentages of times that a node was supported in 100 bootstrap pseudoreplications implemented in ORTHOSCOPE. The scale bar indicates an evolutionary distance of 0.2 substitutions per position; Figure S2: Molecular phylogenetic tree estimated using ORTHOSCOPE with human *PDIA6* as the query sequence (highlighted in purple). Sequences highlighted in blue indicate human orthologs. Those highlighted in pink represent habu (*P. flavoviridis*) orthologs, and those in red boxes represent other snake species. Values beside branches represent percentages of times that a node was supported in 100 bootstrap pseudoreplications implemented in ORTHOSCOPE. The scale bar indicates an evolutionary distance of 0.2 substitutions per position; Figure S3: Molecular phylogenetic tree estimated using ORTHOSCOPE with human *ERP27* as the query sequence (highlighted in purple). Sequences highlighted in blue indicate human orthologs. Those highlighted in pink represent habu (*P. flavoviridis*) orthologs, and those in red boxes represent other snake species. Values beside branches represent percentages of times that a node was supported in 100 bootstrap pseudoreplications implemented in ORTHOSCOPE. The scale bar indicates an evolutionary distance of 0.2 substitutions per position; Figure S4: Molecular phylogenetic tree estimated using ORTHOSCOPE with human *ERP29* as the query sequence (highlighted in purple). Sequences highlighted in blue indicate human orthologs. Those highlighted in pink represent habu (*P. flavoviridis*) orthologs, and those in red boxes represent other snake species. Values beside branches represent percentages of times that a node was supported in 100 bootstrap pseudoreplications implemented in ORTHOSCOPE. The scale bar indicates an evolutionary distance of 0.1 substitutions per position; Figure S5: Molecular phylogenetic tree estimated using ORTHOSCOPE with human *ERP44* as the query sequence (highlighted in purple). Sequences highlighted in blue indicate human orthologs. Those highlighted in pink represent habu (*P. flavoviridis*) orthologs, and those in red boxes represent other snake species. Values beside branches represent percentages of times that a node was supported in 100 bootstrap pseudoreplications implemented in ORTHOSCOPE. The scale bar indicates an evolutionary distance of 0.1 substitutions per position; Figure S6: Molecular phylogenetic tree estimated using ORTHOSCOPE with human *AGR2* as the query sequence (highlighted in purple). Sequences highlighted in blue indicate human orthologs. Those highlighted in pink represent habu (*P. flavoviridis*) orthologs, and those in red boxes represent other snake species. Values beside branches represent percentages of times that a node was supported in 100 bootstrap pseudoreplications implemented in ORTHOSCOPE. The scale bar indicates an evolutionary distance of 0.2 substitutions per position; Figure S7: Molecular phylogenetic tree estimated using ORTHOSCOPE with human *AGR3* as the query sequence (highlighted in purple). Sequences highlighted in blue indicate human orthologs. Those highlighted in pink represent habu (*P. flavoviridis*) orthologs, and those in red boxes represent other snake species. Values beside branches represent percentages of times that a node was supported in 100 bootstrap pseudoreplications implemented in ORTHOSCOPE. The scale bar indicates an evolutionary distance of 0.09 substitutions per position; Figure S8: Molecular phylogenetic tree estimated using ORTHOSCOPE with human *TMX1* as the query sequence (highlighted in purple). Sequences highlighted in blue indicate human orthologs. Those highlighted in pink represent habu (*P. flavoviridis*) orthologs, and those in red boxes represent other snake species. Values beside branches represent percentages of times that a node was supported in 100 bootstrap pseudoreplications implemented in ORTHOSCOPE. The scale bar indicates an evolutionary distance of 0.2 substitutions per position; Figure S9: Molecular phylogenetic tree estimated using ORTHOSCOPE with human *TMX2* as the query sequence (highlighted in purple). Sequences highlighted in blue indicate human orthologs. Those highlighted in pink represent habu (*P. flavoviridis*) orthologs, and those in red boxes represent other snake species. Values beside branches represent percentages of times that a node was supported in 100 bootstrap pseudoreplications implemented in ORTHOSCOPE. The scale bar indicates an evolutionary distance of 0.2 substitutions per position; Figure S10: Molecular phylogenetic tree estimated using ORTHOSCOPE with human *TMX3* as the query sequence (highlighted in purple). Sequences highlighted in blue indicate human orthologs. Those highlighted in pink represent habu (*P. flavoviridis*) orthologs, and those in red boxes represent other snake species. Values beside branches represent percentages of times that a node was supported in 100 bootstrap pseudoreplications implemented in ORTHOSCOPE. The scale bar indicates an evolutionary distance of 0.1 substitutions per position; Figure S11: Molecular phylogenetic tree estimated using ORTHOSCOPE with human *TMX4* as the query sequence (highlighted in purple). Sequences highlighted in blue indicate human orthologs. Those highlighted in pink represent habu (*P. flavoviridis*) orthologs, and those in red boxes represent other snake species. Values beside branches represent percentages of times that a node was supported in 100 bootstrap pseudoreplications implemented in ORTHOSCOPE. The scale bar indicates an evolutionary distance of 0.2 substitutions per position; Figure S12: Molecular phylogenetic tree estimated using ORTHOSCOPE with human *TXNDC5* as the query sequence (highlighted in purple). Sequences highlighted in blue indicate human orthologs. Those highlighted in pink represent habu (*P. flavoviridis*) orthologs, and those in red boxes represent other snake species. Values beside branches represent percentages of times that a node was supported in 100 bootstrap pseudoreplications implemented in ORTHOSCOPE. The scale bar indicates an evolutionary distance of 0.2 substitutions per position; Figure S13: Molecular phylogenetic tree estimated using ORTHOSCOPE with human *TXNDC12* as the query sequence (highlighted in purple). Sequences highlighted in blue indicate human orthologs. Those highlighted in pink represent habu (*P. flavoviridis*) orthologs, and those in red boxes represent other snake species. Values beside branches represent percentages of times that a node was supported in 100 bootstrap pseudoreplications implemented in ORTHOSCOPE. The scale bar indicates an evolutionary distance of 0.2 substitutions per position; Figure S14: Molecular phylogenetic tree estimated using ORTHOSCOPE with human *CASQ1* as the query sequence (highlighted in purple). Sequences highlighted in blue indicate human orthologs. Those highlighted in pink represent habu (*P. flavoviridis*) orthologs, and those in red boxes represent other snake species. Values beside branches represent percentages of times that a node was supported in 100 bootstrap pseudoreplications implemented in ORTHOSCOPE. The scale bar indicates an evolutionary distance of 0.2 substitutions per position; Figure S15: Molecular phylogenetic tree estimated using ORTHOSCOPE with human *CASQ2* as the query sequence (highlighted in purple). Sequences highlighted in blue indicate human orthologs. Those highlighted in pink represent habu (*P. flavoviridis*) orthologs, and those in red boxes represent other snake species. Values beside branches represent percentages of times that a node was supported in 100 bootstrap pseudoreplications implemented in ORTHOSCOPE. The scale bar indicates an evolutionary distance of 0.2 substitutions per position; Figure S16: Molecular phylogenetic tree estimated using ORTHOSCOPE with human *CALR3* as the query sequence (highlighted in purple). Sequences highlighted in blue indicate human orthologs. Those highlighted in pink represent habu (*P. flavoviridis*) orthologs, and those in red boxes represent other snake species. Values beside branches represent percentages of times that a node was supported in 100 bootstrap pseudoreplications implemented in ORTHOSCOPE. The scale bar indicates an evolutionary distance of 0.2 substitutions per position; Figure S17: Molecular phylogenetic tree estimated using ORTHOSCOPE with human *CANX* as the query sequence (highlighted in purple). Sequences highlighted in blue indicate human orthologs. Those highlighted in pink represent habu (*P. flavoviridis*) orthologs, and those in red boxes represent other snake species. Values beside branches represent percentages of times that a node was supported in 100 bootstrap pseudoreplications implemented in ORTHOSCOPE. The scale bar indicates an evolutionary distance of 0.2 substitutions per position; Figure S18: Molecular phylogenetic tree estimated using ORTHOSCOPE with *Danio rerio CALRL* as the query sequence (highlighted in purple). Sequences highlighted in blue indicate human orthologs. Those highlighted in pink represent habu (*P. flavoviridis*) orthologs, and those in red boxes represent other snake species. Values beside branches represent percentages of times that a node was supported in 100 bootstrap pseudoreplications implemented in ORTHOSCOPE. The scale bar indicates an evolutionary distance of 0.2 substitutions per position; Figure S19: *K_A_/K_S_* plot for major venom protein modification candidate enzyme or chaperone genes *P4HB* (left) and *PDIA3* (right). *K_A_* and *K_S_* values were calculated by the Nei-Gojobori method for each pairwise genes: venomous snake vs venomous snake (●), venomous snake vs other animal (including human) (▲), and other animal vs other animal (◯). Venomous snakes include *Protobothrops flavoviridis* and *P. mucrosquamatus*, and other animals include human, mouse, chicken, anole and non-venomous snakes (*Python bivittatus* and *Thamnophis sirtalis*); Figure S20: *K_A_/K_S_* plot for major venom protein modification candidate enzyme or chaperone genes, *CALR* (left) and *SELENOM* (right). *K_A_* and *K_S_* values were calculated by the Nei-Gojobori method for each pairwise genes: venomous snake vs venomous snake (●), venomous snake vs other animals (including human) (▲), and other animals vs other animals (◯). Venomous snakes include *Protobothrops flavoviridis* and *P. mucrosquamatus*, and other animals include human, mouse, chicken, anole and non-venomous snakes (*Python bivittatus* and *Thamnophis sirtalis*); Table S1: Genes highly expressed in venom glands from the first adult habu (venom gland-specimen#1: a sub-adult specimen).
