# Supplementary material for: Active Expression of Genes for Protein Modification Enzymes in Habu Venom Glands
Source: Toxins (Basel). 2022 Apr 22;14(5):300. doi: 10.3390/toxins14050300 (PMC9146206; doi:10.3390/toxins14050300)
Supplement: Supplementary file 1 [file toxins-14-00300-s001.zip › toxins-1576650-supplementary.pdf]

Supplementary Materials: Active Expression of Genes for Protein Modification Enzymes in Habu Venom Glands

Akiko Isomoto, Eiichi Shoguchi, Kanako Hisata, Jun Inoue, Yinrui Sun, Kenji Inaba, Noriyuki Satoh, Tomohisa Ogawa and Hiroki Shibata

Table S1. Genes highly expressed in venom glands from the first adult habu (venom gland-specimen#1: a sub-adult specimen).

Gene Model ID	Gene name	Annotation by Blast2GO	TPM	
habu1_s258676_g24318.t1	*CNP01*	bradykinin-potentiating and C-type natriuretic peptides	260,950.0	Venom protein
habu1_s14911_g21429.t1	*svMP06*	svMP06, H2, metalloproteinase (metalloprotease P-Iia)	83,772.3	Venom protein
habu1_s47459_g23397.t1	*svPLA2-02, svPLA2-03*	svPLA2-02, PL-Y/PL-X'/PL-B, svPLA2-03, PLA-N	24,153.9	Venom protein
habu1_s14911_g21430.t1	*svMP07*	svMP07, HR2a, svMP08, flavoridin	19,406.5	Venom protein
habu1_s565_g02677.t1		creatine kinase m-type	13,375.4	
habu1_s3443_g11710.t1	*P4HB*	protein disulfide-isomerase	13,303.9	Modification enzyme for proteins analyzed in this study
habu1_s10061_g19809.t1	*svCTLP06*	svCTLP06, flavocetin-A beta	13,143.3	Venom protein
habu1_s9571_g19434.t1	*svPLA2-01*	svPLA2-01, Asp49PLA2/PL1a/PL2a	9,325.0	Venom protein
habu1_s9571_g19433.t1	*svPLA2-08*	svPLA2-08, PfPLA6	8,532.3	Venom protein
habu1_s2592_g09356.t1		parvalbumin beta-like	8,060.9	
habu1_s1877_g06936.t1		myosin light chain	7,569.0	
habu1_s4277_g13818.t1		alpha skeletal muscle	7,092.6	
habu1_s301_g01413.t1		PREDICTED: troponin T, fast skeletal muscle isoforms-like isoform X7, X5 [Python bivittatus]	6,268.5	
habu1_s10061_g19810.t1	*svCTLP03, svCTLP04, svCTLP07*	svCTLP07 rhodocetinEMS16-like-beta	6,259.5	Venom protein
habu1_s867_g03056.t1		calglandulin-like protein	6,243.7	
habu1_s158_g00895.t1		forkhead box protein j1	5,899.5	
habu1_s9570_g19432.t1	*svPLA2-07*	svPLA2-07 PL1b/PL2b	5,853.2	Venom protein
habu1_s5309_g15576.t1		PREDICTED: 40S ribosomal protein, S25	5,337.2	Ribosomal protein
habu1_s2087_g07635.t1		ribosomal protein	5,084.4	Ribosomal protein
habu1_s4158_g13542.t1		55 kda erythrocyte membrane protein,	5,003.6	
habu1_s1917_g07050.t1		60s ribosomal protein l7a	4,207.0	Ribosomal protein

habu1_s9530_g19385.t1		PREDICTED: elongation factor 1-beta	4,185.3	
habu1_s2955_g10552.t1		elongation factor 1-alpha 1	4,094.6	
habu1_s1466_g05403.t1		peptidyl-proryl cis-trans isomerase a-like	4,091.7	
habu1_s16120_g21649.t1		60s ribosomal protein l18a	4,042.8	Ribosomal protein
habu1_s68614_g23647.t1		60s ribosomal protein l7	4,037.2	Ribosomal protein
habu1_s7991_g18513.t1		40s ribosomal protein partial	3,958.8	Ribosomal protein
habu1_s7978_g18472.t1		60s acidic ribosomal protein p2	3,796.5	Ribosomal protein
habu1_s2370_g08604.t1		calsquestrin-partial	3,732.7	
habu1_s6274_g17028.t1		ubiquitin-40s ribosomal protein s27a	3,642.4	
habu1_s3168_g10977.t1	*svCTLP08*	svCTLP08, C-lectin02	3,625.6	Venom protein
habu1_s3258_g11210.t1	*svMP04*	svMP04, jerdonitin-like	3,623.7	Venom protein
habu1_s3258_g11211.t1	*svMP05*	svMP05, HR1a	3,603.2	Venom protein
habu1_s7284_g17906.t1		40s ribosomal protein s13	3,532.7	Ribosomal protein
habu1_s37466_g23178.t1		growth-related translationally controlled tumor protein	3,513.5	
habu1_s74_g00428.t1		60s ribosomal protein l11	3,486.1	Ribosomal protein
habu1_s2449_g08987.t1		60s ribosomal protein l31	3,393.7	Ribosomal protein
habu1_s3765_g12603.t1		40s ribosomal protein s3a	3,384.6	Ribosomal protein
habu1_s86658_g23820.t1		60s acidic ribosomal protein p1	3,142.2	Ribosomal protein
habu1_s402938_g24930.t1		60s ribosomal protein l36	2,914.9	Ribosomal protein
habu1_s2592_g09366.t1		cytoplasmic 2	2,911.7	
habu1_s1924_g07069.t1		40s ribosomal protein s15a	2,909.6	Ribosomal protein
habu1_s2444_g08904.t1		60s ribosomal protein l30	2,907.9	Ribosomal protein
habu1_s1408_g05043.t1		60s ribosomal protein l5	2,888.9	Ribosomal protein
habu1_s1529_g05741.t1		40s ribosomal protein s10	2,740.9	Ribosomal protein
habu1_s188_g01057.t1		60s ribosomal protein partial	2,630.9	Ribosomal protein
habu1_s4331_g13956.t1		40s ribosomal protein s8	2,598.8	Ribosomal protein
habu1_s3872_g12862.t1		40s ribosomal protein s20	2,572.6	Ribosomal protein
habu1_s231_g01271.t1		60s ribosomal protein l24	2,569.3	Ribosomal protein
habu1_s336341_g24425.t1		claudin- partial	2,558.1	

Transcripts are expressed as "Transcripts per million" (TPM). red: Venom protein genes, blue: Protein modification genes analyzed in this study.

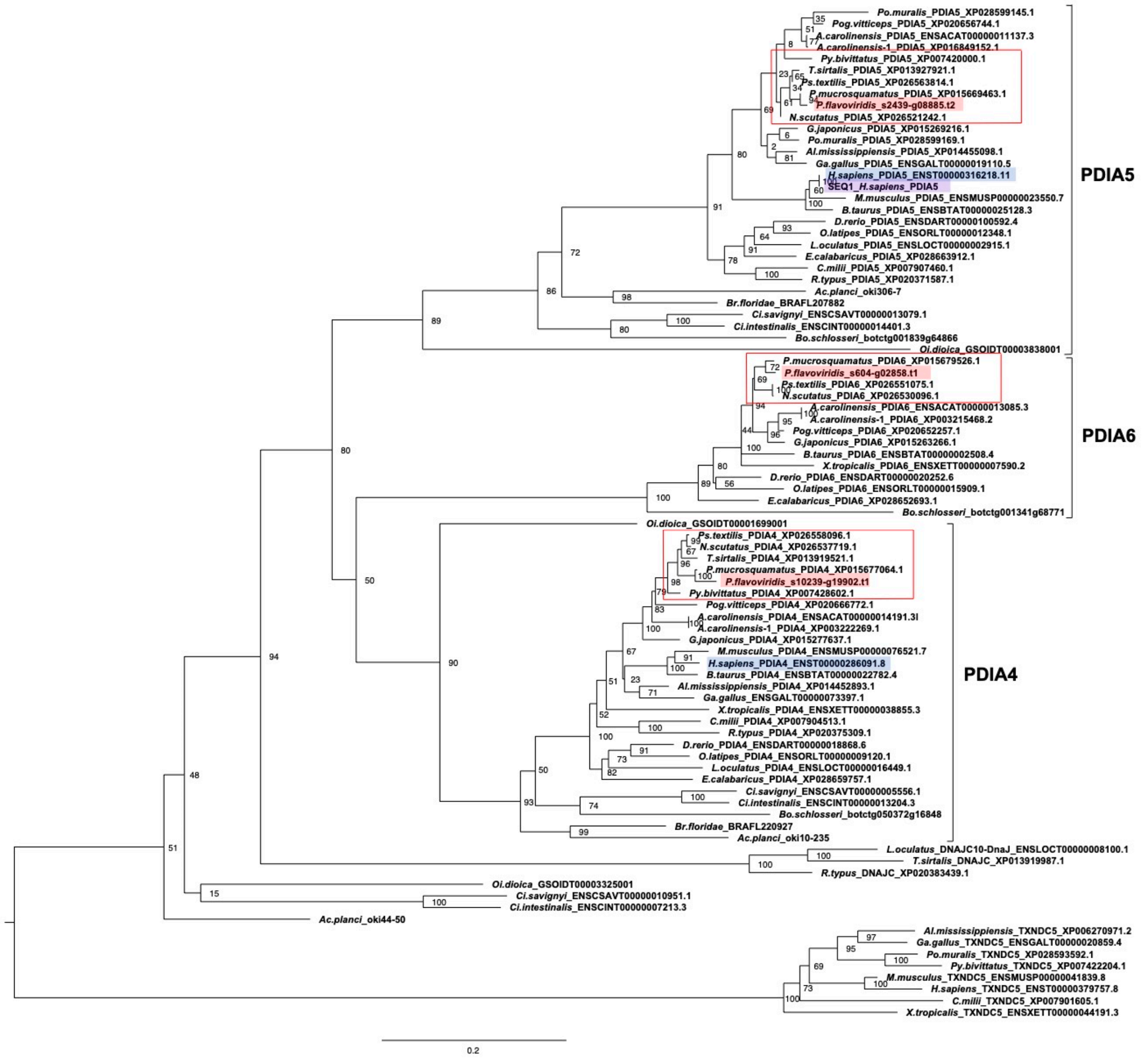

Figure S1. Molecular phylogenetic tree estimated using ORTHOSCOPE with human *PDIA5* as the query sequence (highlighted in purple).

Sequences highlighted in blue indicate human orthologs. Those highlighted in pink represent habu (*P. flavoviridis*) orthologs, and those in red boxes represent other snake species. Values beside branches represent percentages of times that a node was supported in 100 bootstrap pseudoreplications implemented in ORTHOSCOPE. The scale bar indicates an evolutionary distance of 0.2 substitutions per position.

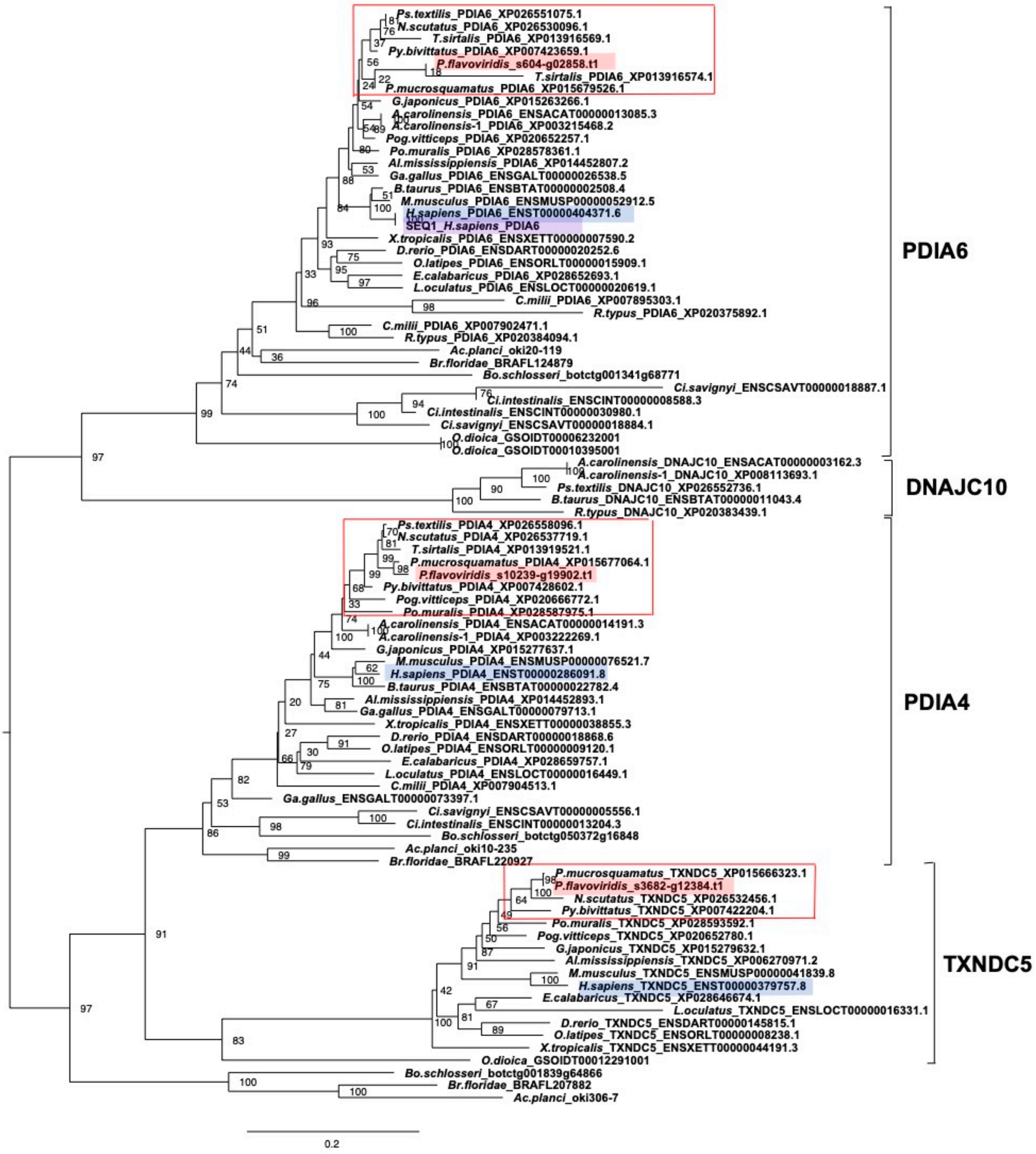

Figure S2. Molecular phylogenetic tree estimated using ORTHOSCOPE with human *PDIA6* as the query sequence (highlighted in purple).

Sequences highlighted in blue indicate human orthologs. Those highlighted in pink represent habu (*P. flavoviridis*) orthologs, and those in red boxes represent other snake species. Values beside branches represent percentages of times that a node was supported in 100 bootstrap pseudoreplications implemented in ORTHOSCOPE. The scale bar indicates an evolutionary distance of 0.2 substitutions per position.

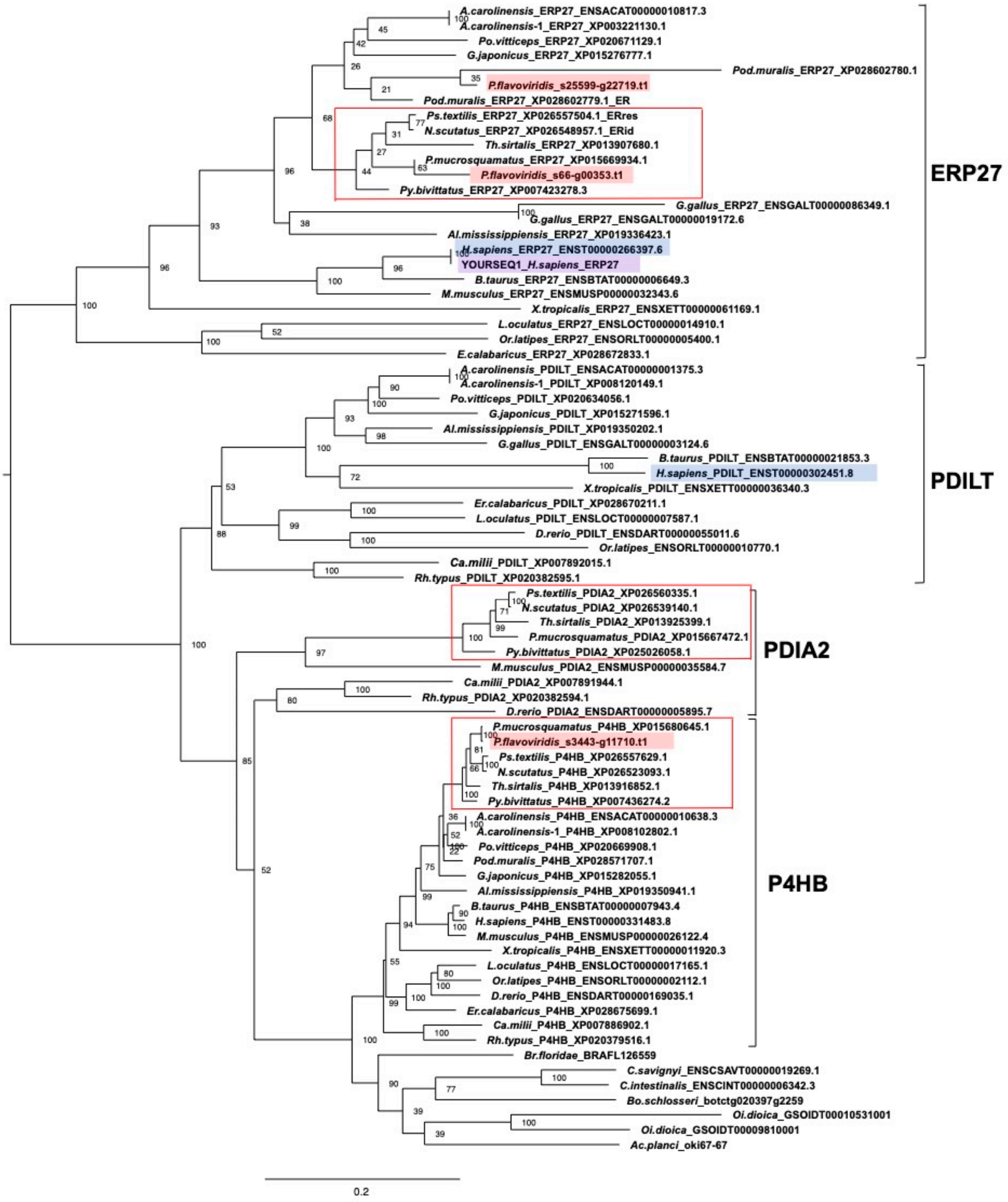

Figure S3. Molecular phylogenetic tree estimated using ORTHOSCOPE with human *ERP27* as the query sequence (highlighted in purple).

Sequences highlighted in blue indicate human orthologs. Those highlighted in pink represent habu (*P. flavoviridis*) orthologs, and those in red boxes represent other snake species. Values beside branches represent percentages of times that a node was supported in 100 bootstrap pseudoreplications implemented in ORTHOSCOPE. The scale bar indicates an evolutionary distance of 0.2 substitutions per position.

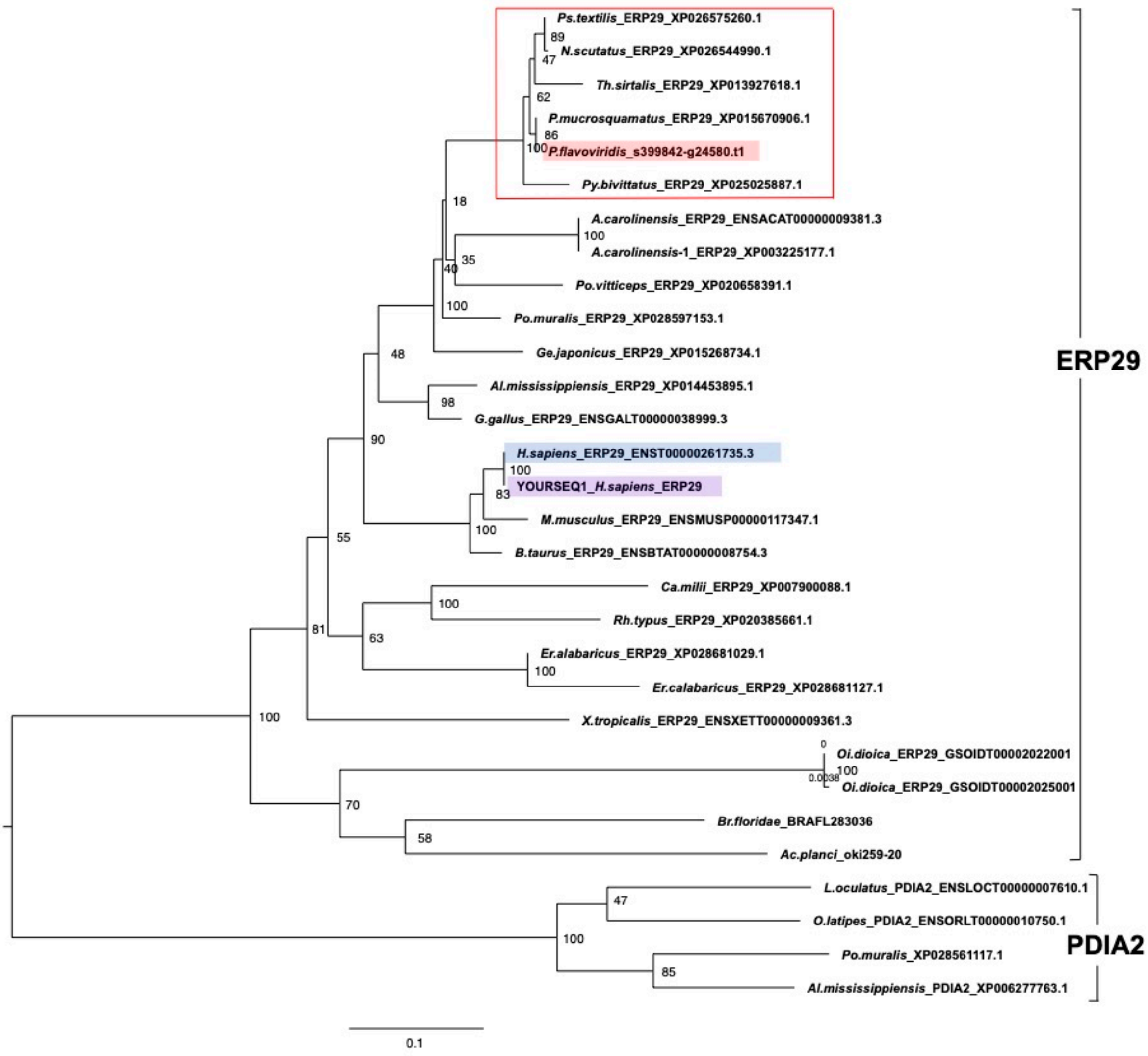

Figure S4. Molecular phylogenetic tree estimated using ORTHOSCOPE with human *ERP29* as the query sequence (highlighted in purple).

Sequences highlighted in blue indicate human orthologs. Those highlighted in pink represent habu (*P. flavoviridis*) orthologs, and those in red boxes represent other snake species. Values beside branches represent percentages of times that a node was supported in 100 bootstrap pseudoreplications implemented in ORTHOSCOPE. The scale bar indicates an evolutionary distance of 0.1 substitutions per position.

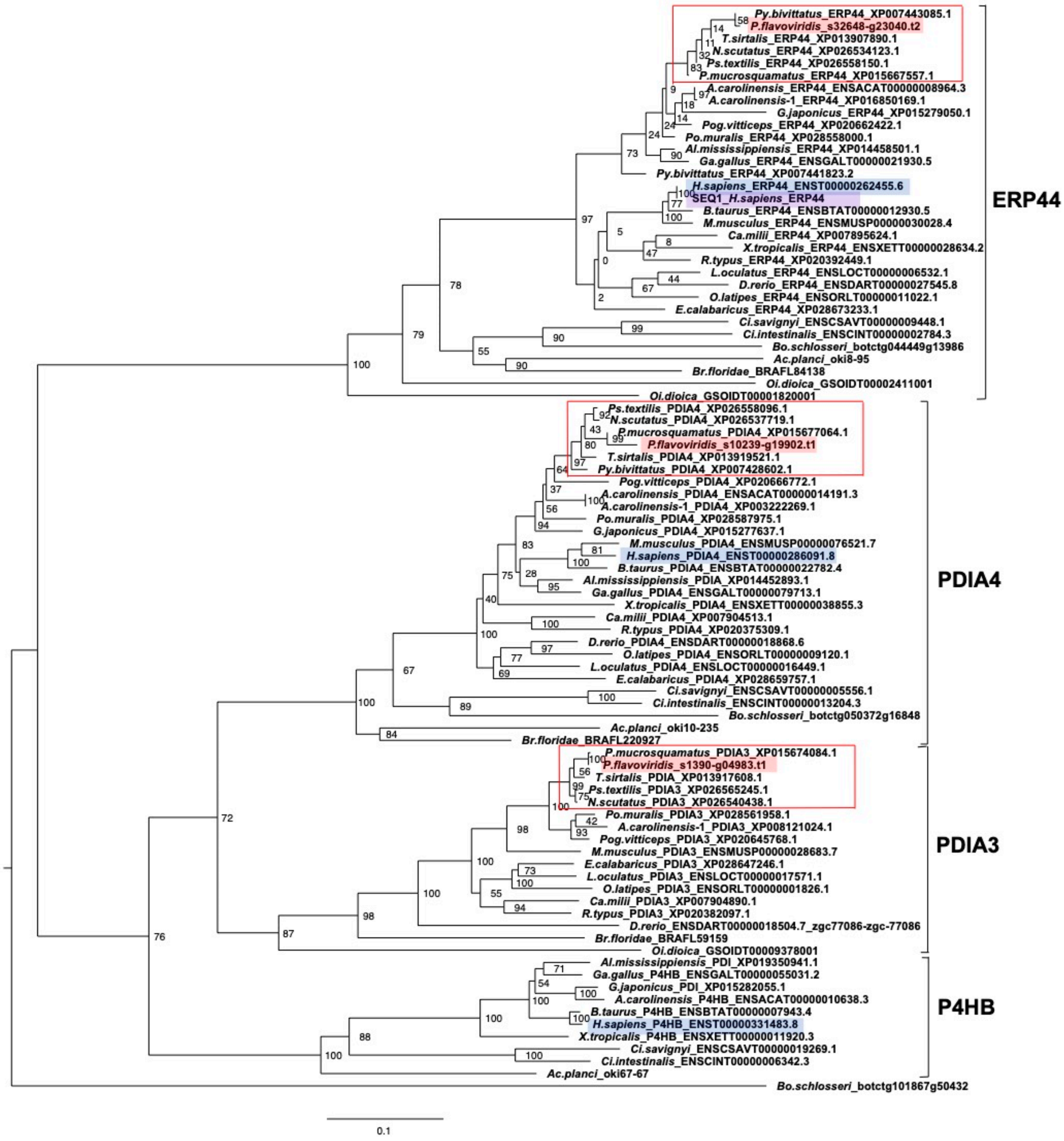

Figure S5. Molecular phylogenetic tree estimated using ORTHOSCOPE with human *ERP44* as the query sequence (highlighted in purple).

Sequences highlighted in blue indicate human orthologs. Those highlighted in pink represent habu (*P. flavoviridis*) orthologs, and those in red boxes represent other snake species. Values beside branches represent percentages of times that a node was supported in 100 bootstrap pseudoreplications implemented in ORTHOSCOPE. The scale bar indicates an evolutionary distance of 0.1 substitutions per position.

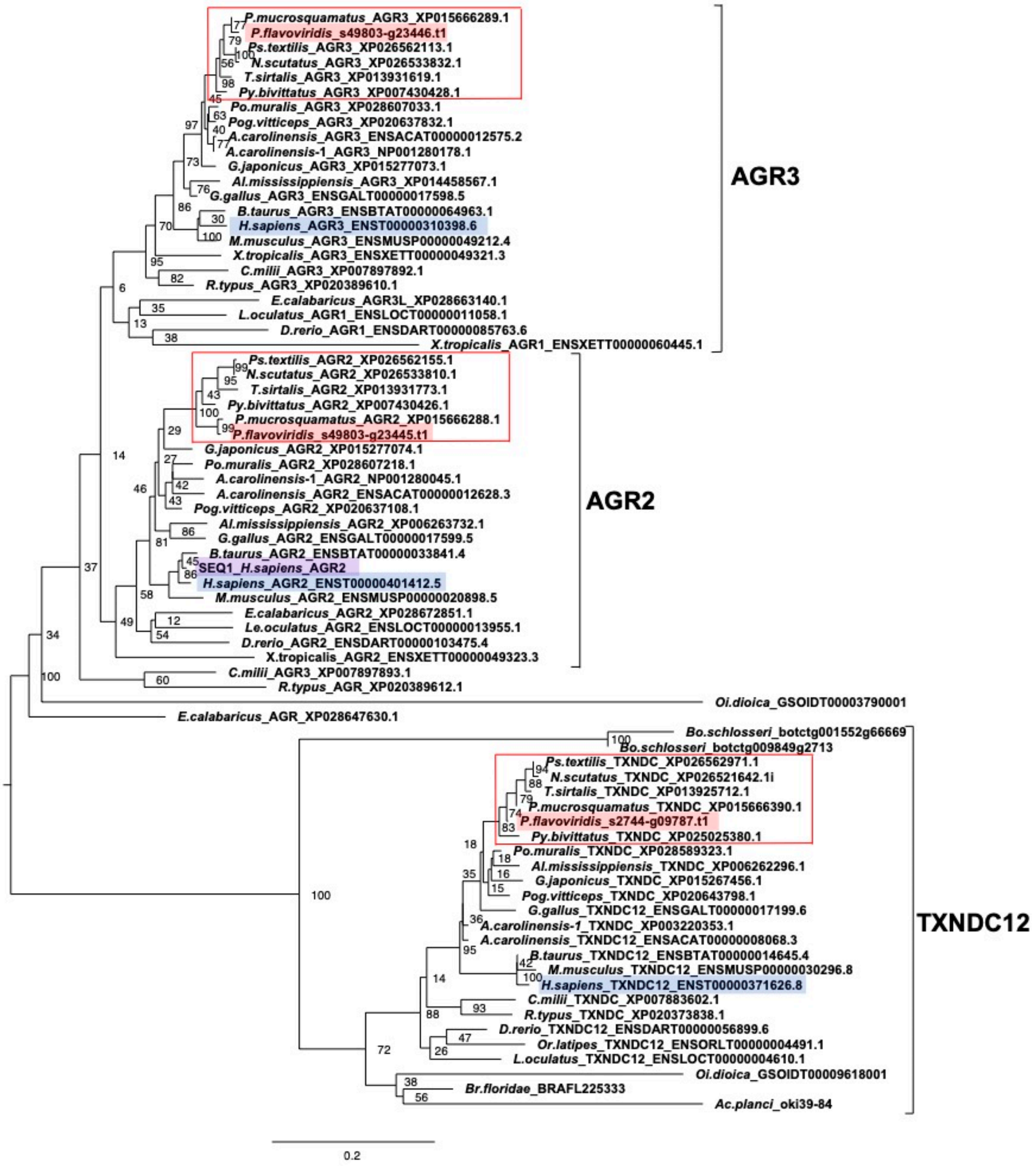

Figure S6. Molecular phylogenetic tree estimated using ORTHOSCOPE with human *AGR2* as the query sequence (highlighted in purple).

Sequences highlighted in blue indicate human orthologs. Those highlighted in pink represent habu (*P. flavoviridis*) orthologs, and those in red boxes represent other snake species. Values beside branches represent percentages of times that a node was supported in 100 bootstrap pseudoreplications implemented in ORTHOSCOPE. The scale bar indicates an evolutionary distance of 0.2 substitutions per position.

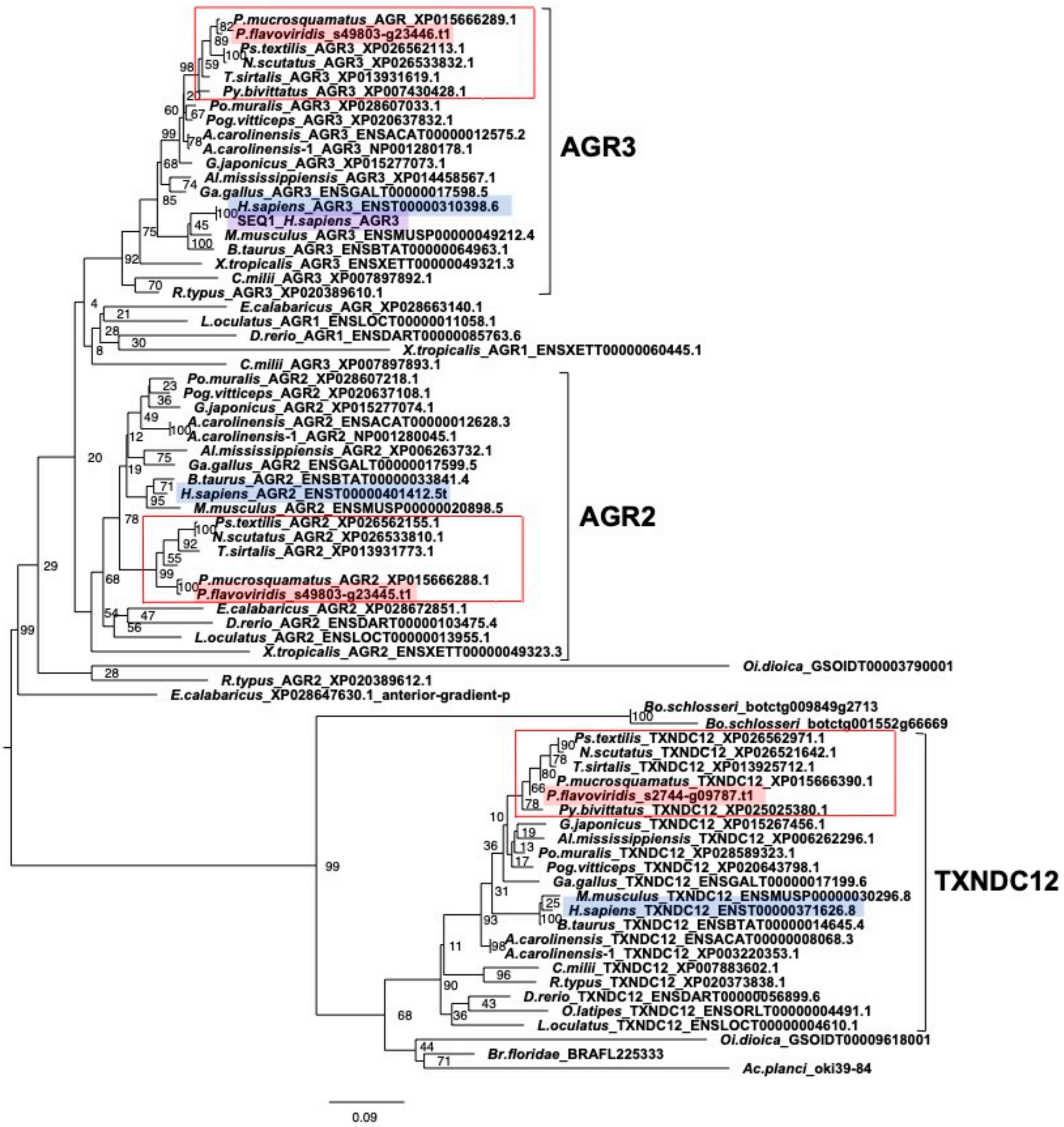

Figure S7. Molecular phylogenetic tree estimated using ORTHOSCOPE with human *AGR3* as the query sequence (highlighted in purple).

Sequences highlighted in blue indicate human orthologs. Those highlighted in pink represent habu (*P. flavoviridis*) orthologs, and those in red boxes represent other snake species. Values beside branches represent percentages of times that a node was supported in 100 bootstrap pseudoreplications implemented in ORTHOSCOPE. The scale bar indicates an evolutionary distance of 0.09 substitutions per position.

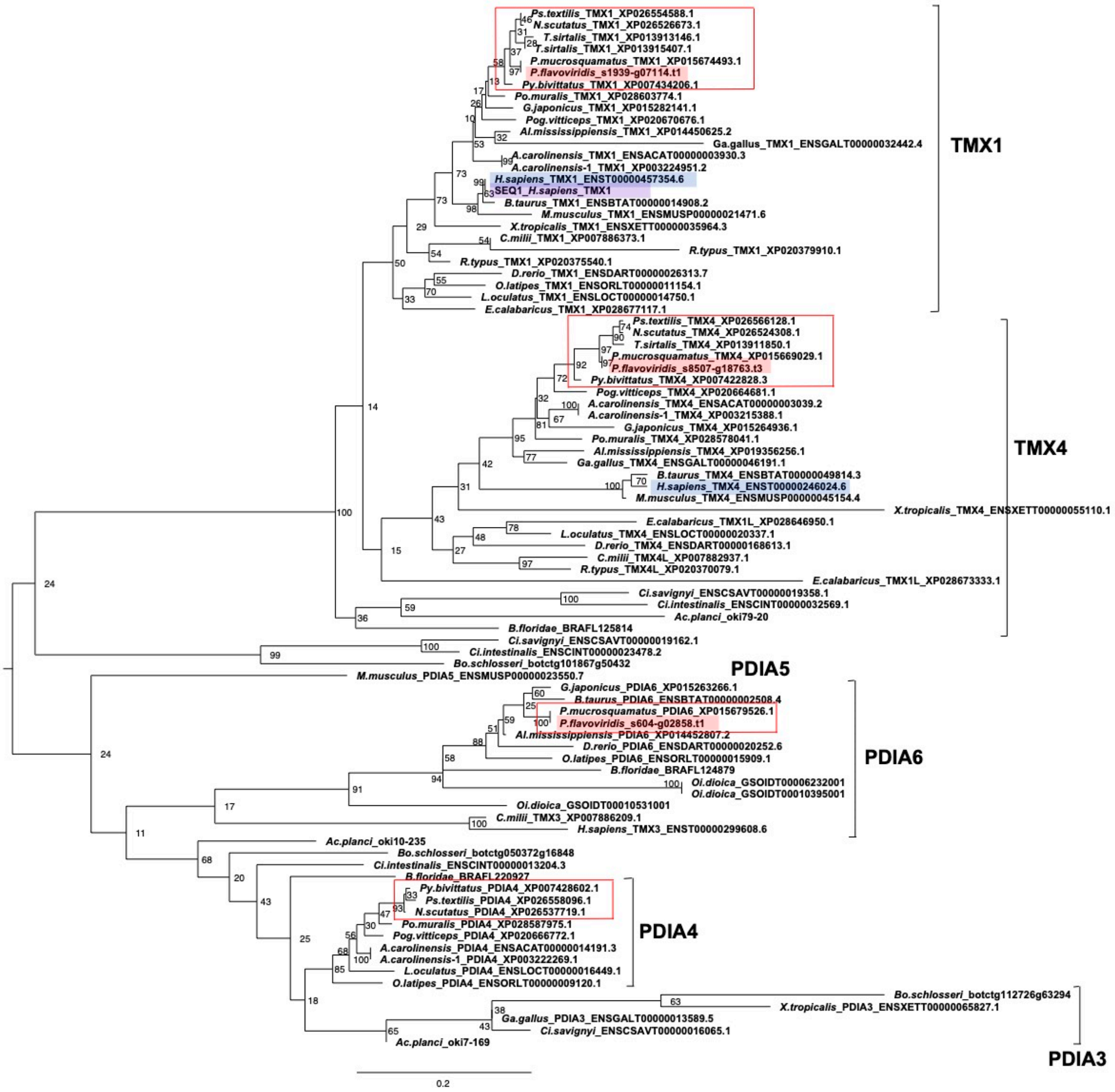

Figure S8. Molecular phylogenetic tree estimated using ORTHOSCOPE with human *TMX1* as the query sequence (highlighted in purple).

Sequences highlighted in blue indicate human orthologs. Those highlighted in pink represent habu (*P. flavoviridis*) orthologs, and those in red boxes represent other snake species. Values beside branches represent percentages of times that a node was supported in 100 bootstrap pseudoreplications implemented in ORTHOSCOPE. The scale bar indicates an evolutionary distance of 0.2 substitutions per position.

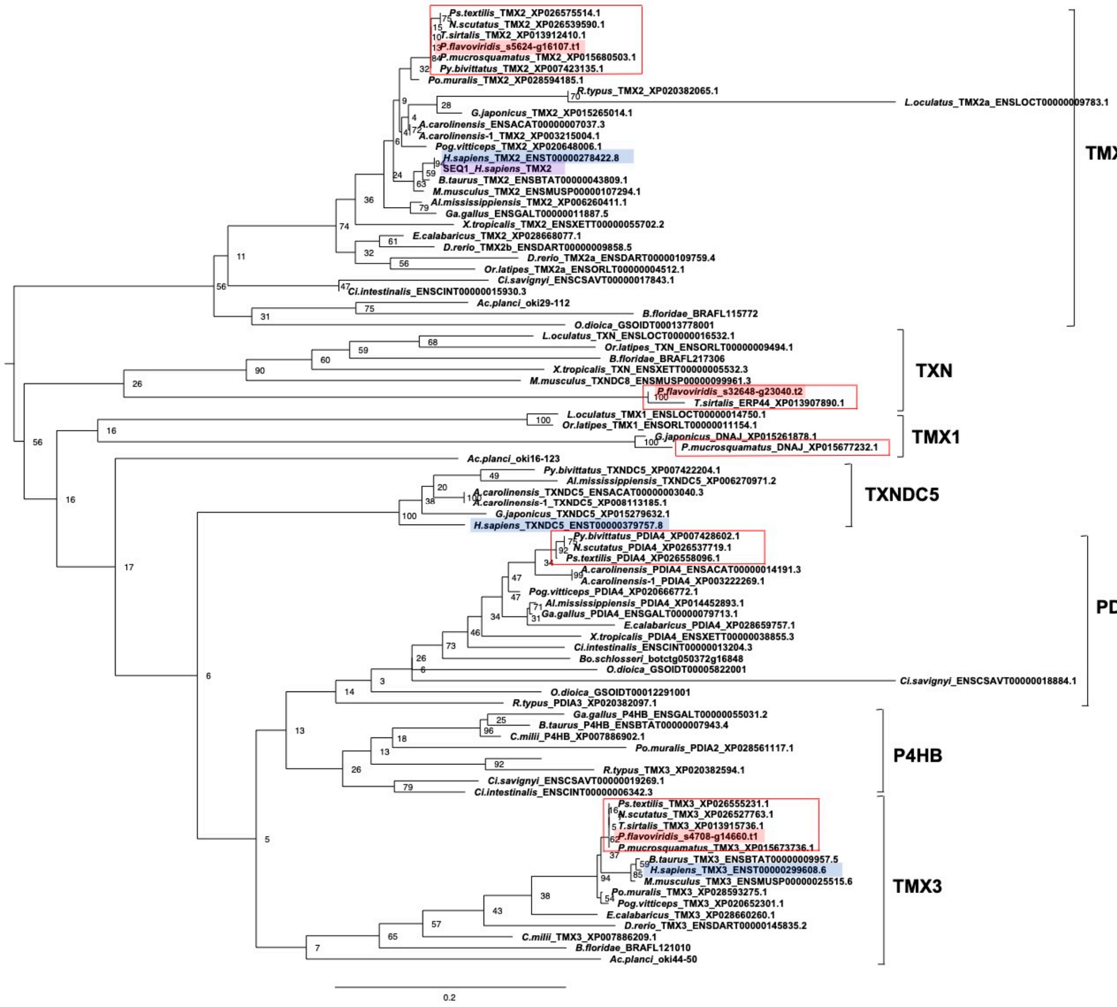

Figure S9. Molecular phylogenetic tree estimated using ORTHOSCOPE with human *TMX2* as the query sequence (highlighted in purple).

Sequences highlighted in blue indicate human orthologs. Those highlighted in pink represent habu (*P. flavoviridis*) orthologs, and those in red boxes represent other snake species. Values beside branches represent percentages of times that a node was supported in 100 bootstrap pseudoreplications implemented in ORTHOSCOPE. The scale bar indicates an evolutionary distance of 0.2 substitutions per position.

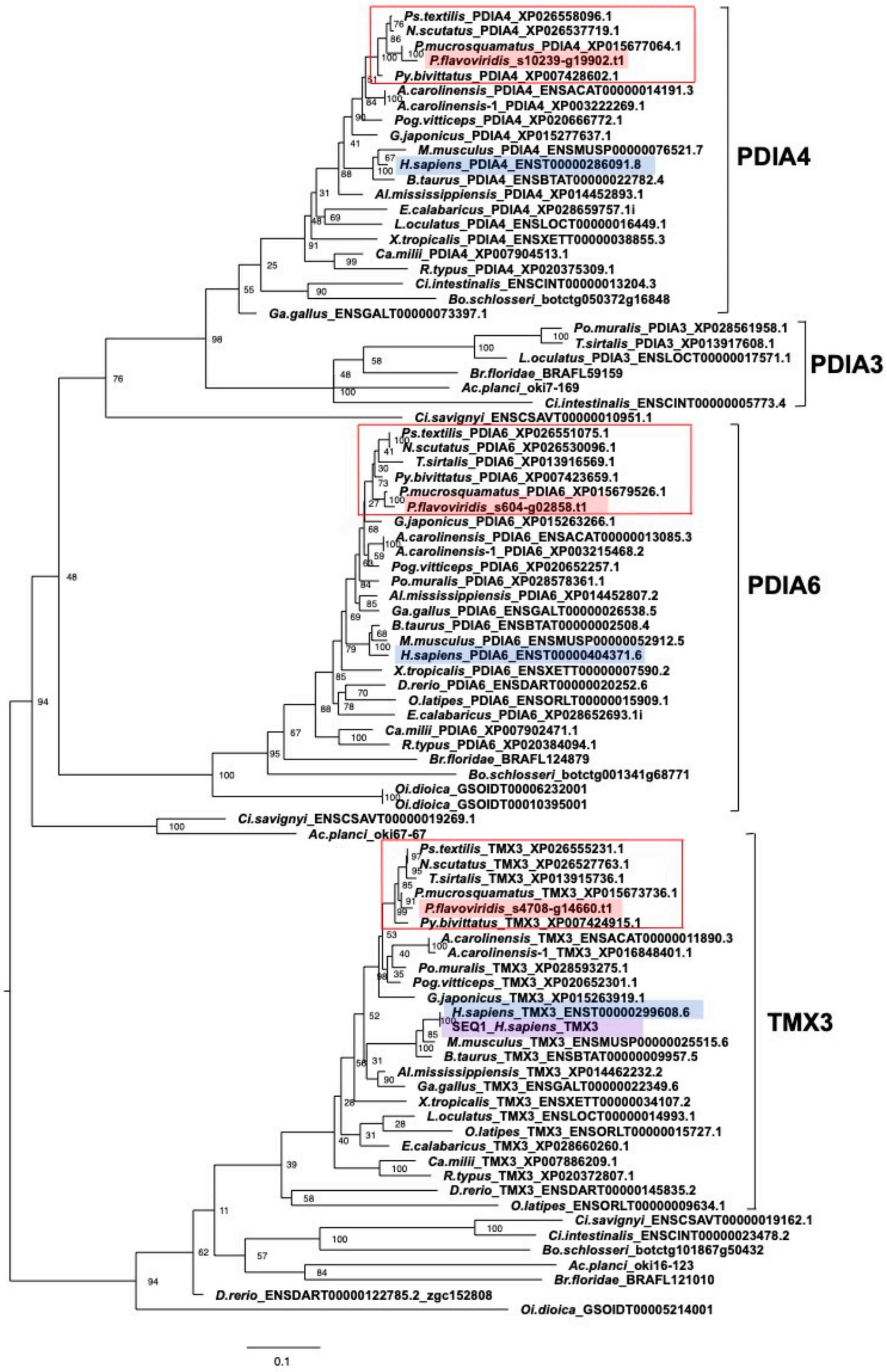

Figure S10. Molecular phylogenetic tree estimated using ORTHOSCOPE with human *TMX3* as the query sequence (highlighted in purple).

Sequences highlighted in blue indicate human orthologs. Those highlighted in pink represent habu (*P. flavoviridis*) orthologs, and those in red boxes represent other snake species. Values beside branches represent percentages of times that a node was supported in 100 bootstrap pseudoreplications implemented in ORTHOSCOPE. The scale bar indicates an evolutionary distance of 0.1 substitutions per position.

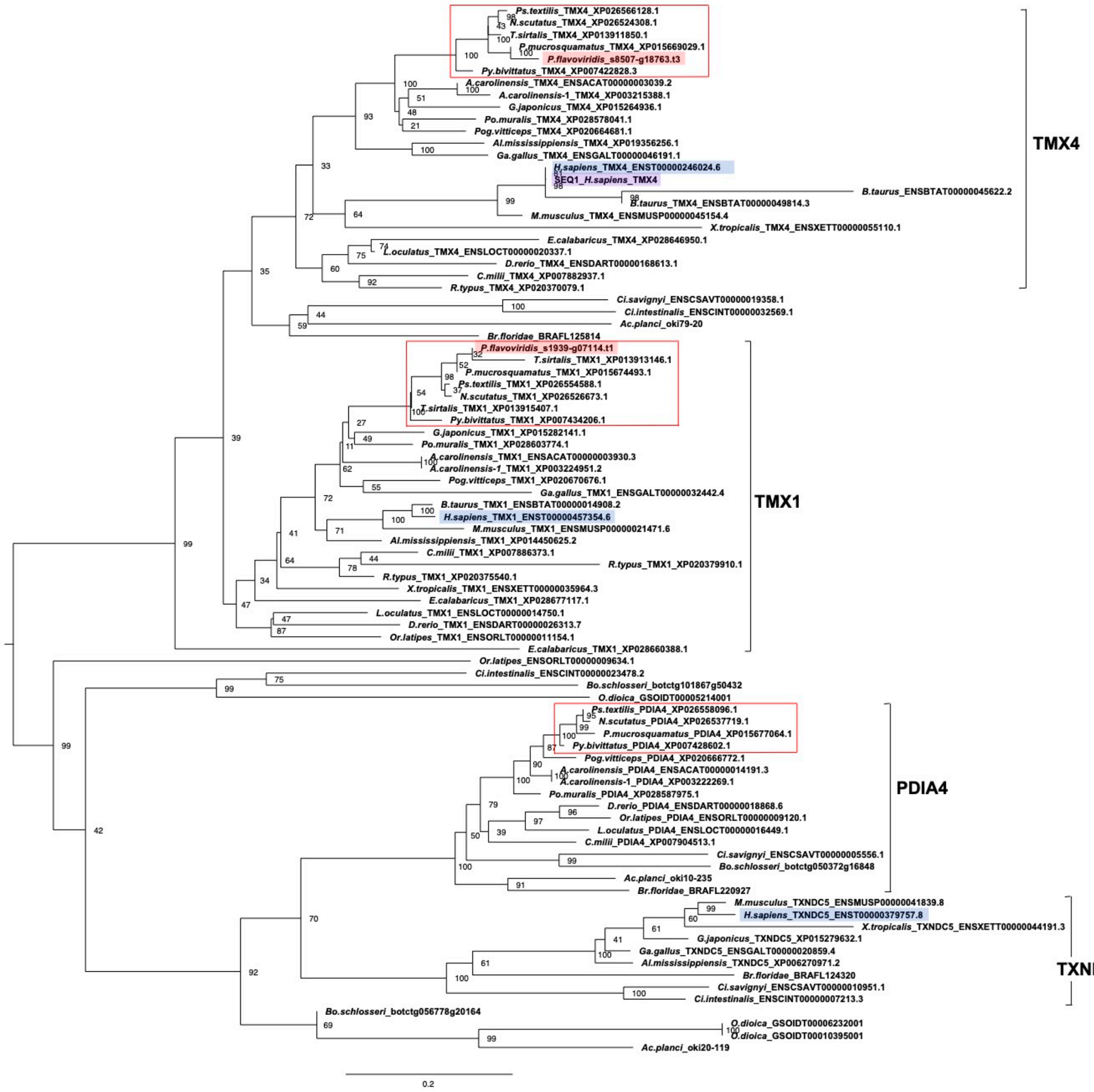

Figure S11. Molecular phylogenetic tree estimated using ORTHOSCOPE with human *TMX4* as the query sequence (highlighted in purple).

Sequences highlighted in blue indicate human orthologs. Those highlighted in pink represent habu (*P. flavoviridis*) orthologs, and those in red boxes represent other snake species. Values beside branches represent percentages of times that a node was supported in 100 bootstrap pseudoreplications implemented in ORTHOSCOPE. The scale bar indicates an evolutionary distance of 0.2 substitutions per position.

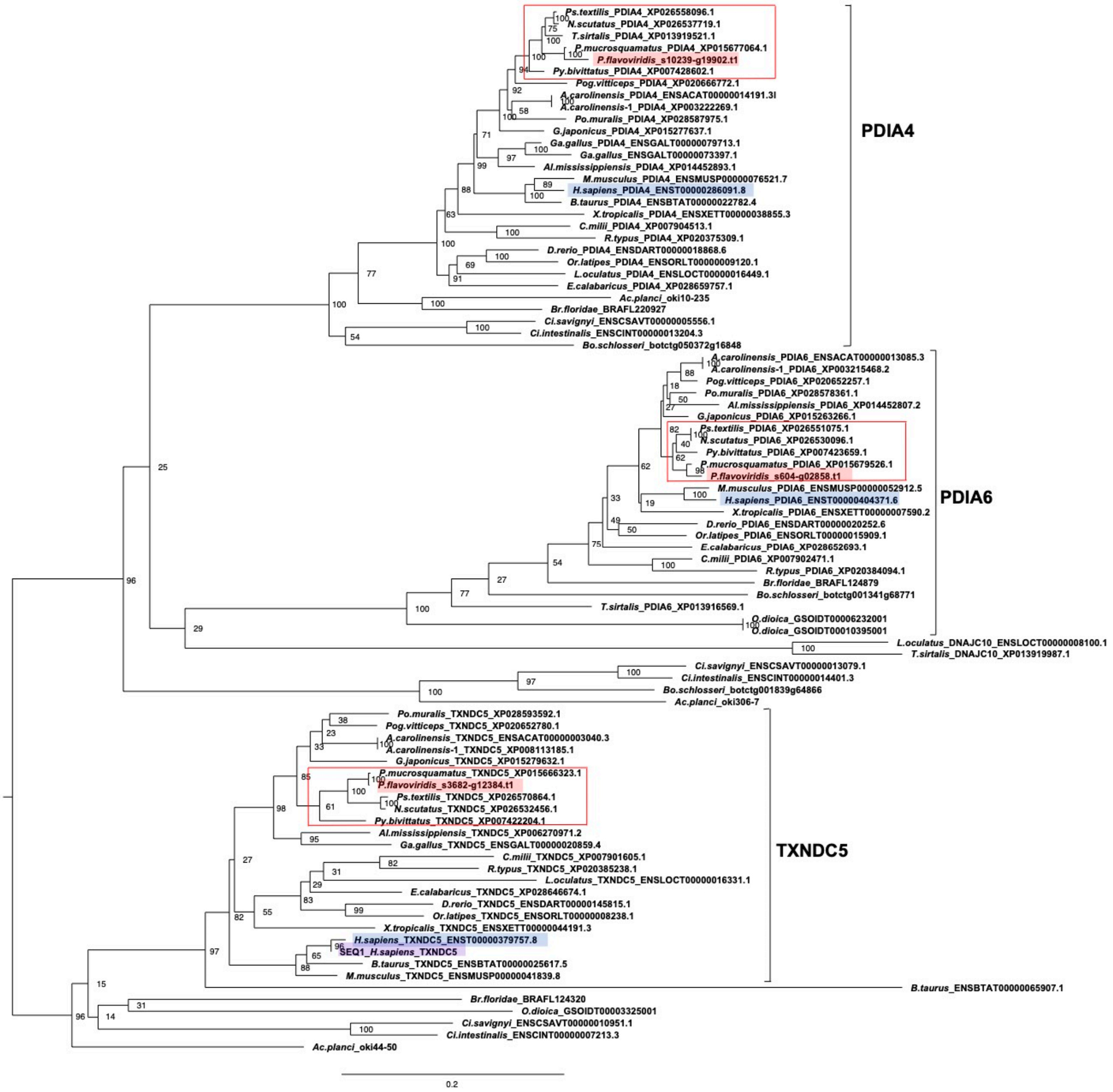

Figure S12. Molecular phylogenetic tree estimated using ORTHOSCOPE with human *TXNDC5* as the query sequence (highlighted in purple).

Sequences highlighted in blue indicate human orthologs. Those highlighted in pink represent habu (*P. flavoviridis*) orthologs, and those in red boxes represent other snake species. Values beside branches represent percentages of times that a node was supported in 100 bootstrap pseudoreplications implemented in ORTHOSCOPE. The scale bar indicates an evolutionary distance of 0.2 substitutions per position.

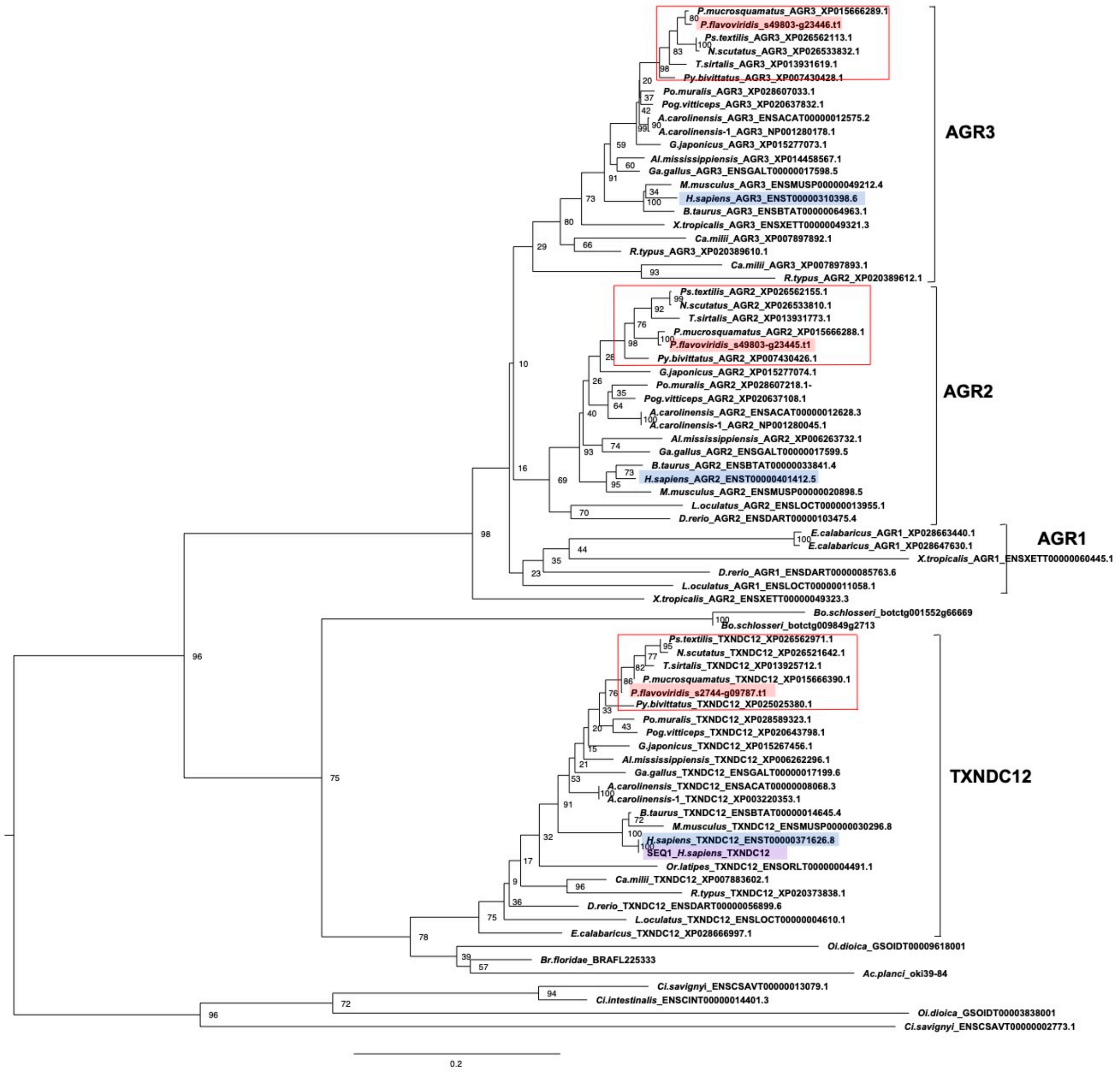

Figure S13. Molecular phylogenetic tree estimated using ORTHOSCOPE with human *TXNDC12* as the query sequence (highlighted in purple).

Sequences highlighted in blue indicate human orthologs. Those highlighted in pink represent habu (*P. flavoviridis*) orthologs, and those in red boxes represent other snake species. Values beside branches represent percentages of times that a node was supported in 100 bootstrap pseudoreplications implemented in ORTHOSCOPE. The scale bar indicates an evolutionary distance of 0.2 substitutions per position.

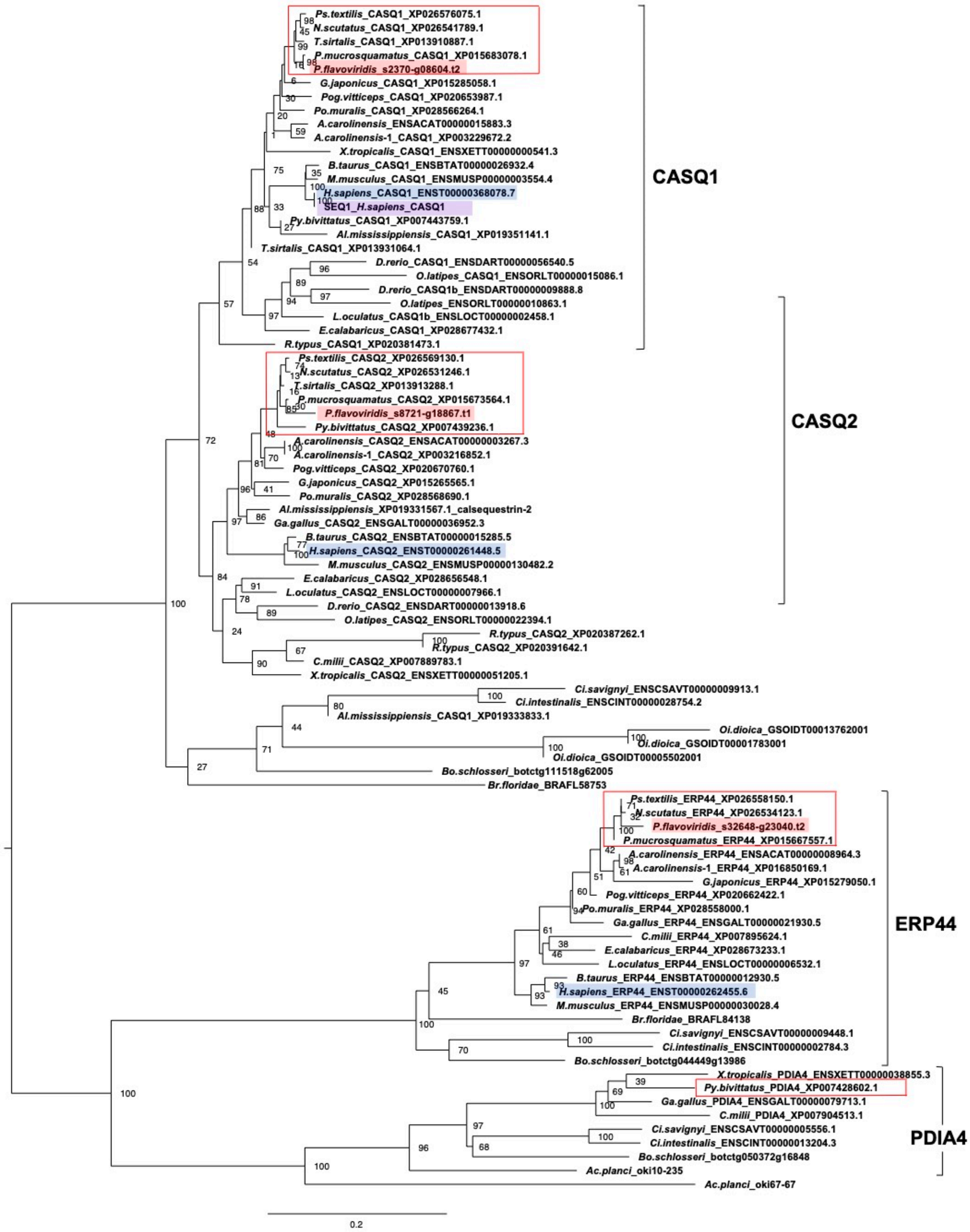

Figure S14. Molecular phylogenetic tree estimated using ORTHOSCOPE with human *CASQ1* as the query sequence (highlighted in purple).

Sequences highlighted in blue indicate human orthologs. Those highlighted in pink represent habu (*P. flavoviridis*) orthologs, and those in red boxes represent other snake species. Values beside branches represent percentages of times that a node was supported in 100 bootstrap pseudoreplications implemented in ORTHOSCOPE. The scale bar indicates an evolutionary distance of 0.2 substitutions per position.

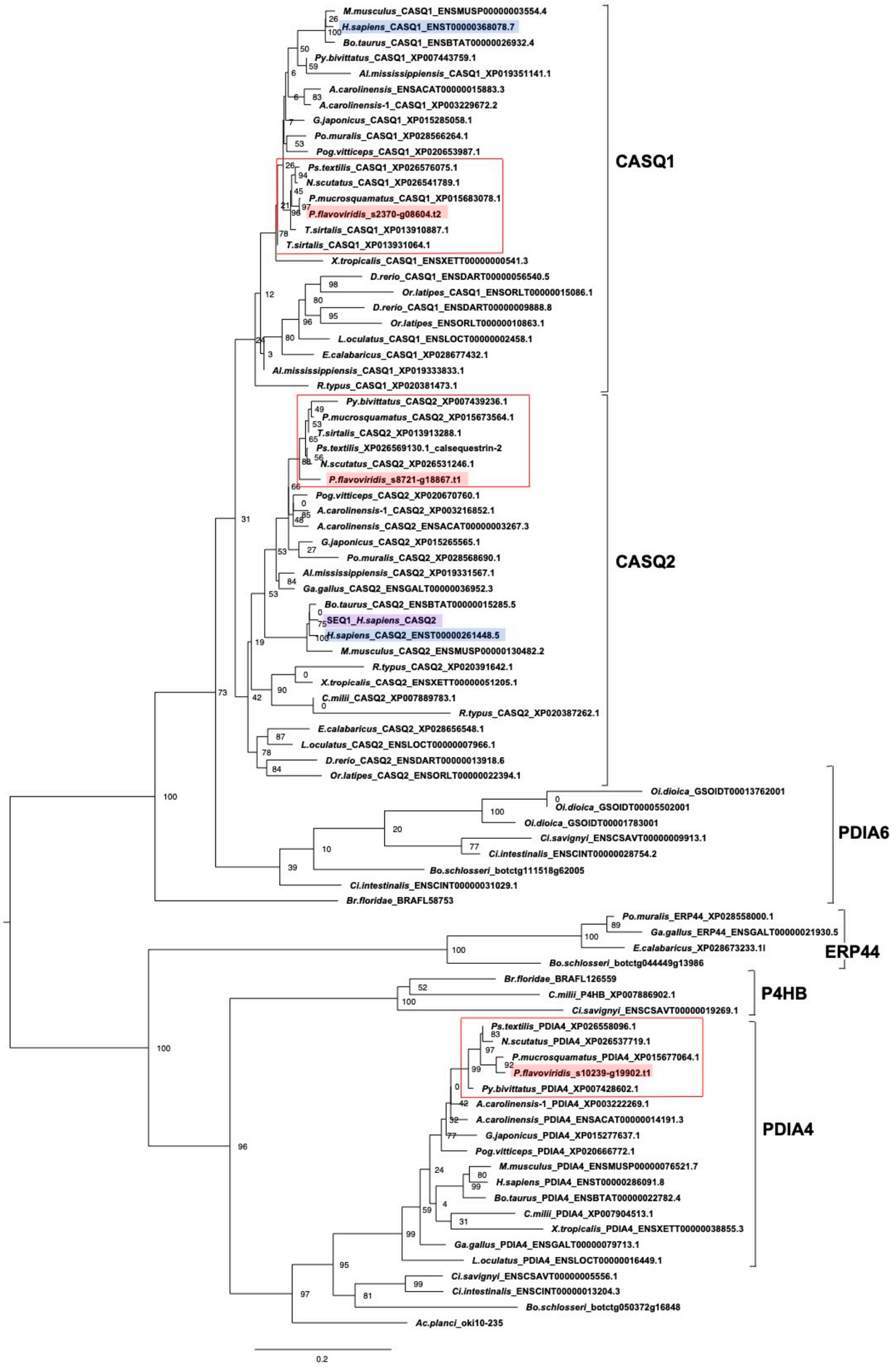

Figure S15. Molecular phylogenetic tree estimated using ORTHOSCOPE with human *CASQ2* as the query sequence (highlighted in purple).

Sequences highlighted in blue indicate human orthologs. Those highlighted in pink represent habu (*P. flavoviridis*) orthologs, and those in red boxes represent other snake species. Values beside branches represent percentages of times that a node was supported in 100 bootstrap pseudoreplications implemented in ORTHOSCOPE. The scale bar indicates an evolutionary distance of 0.2 substitutions per position.

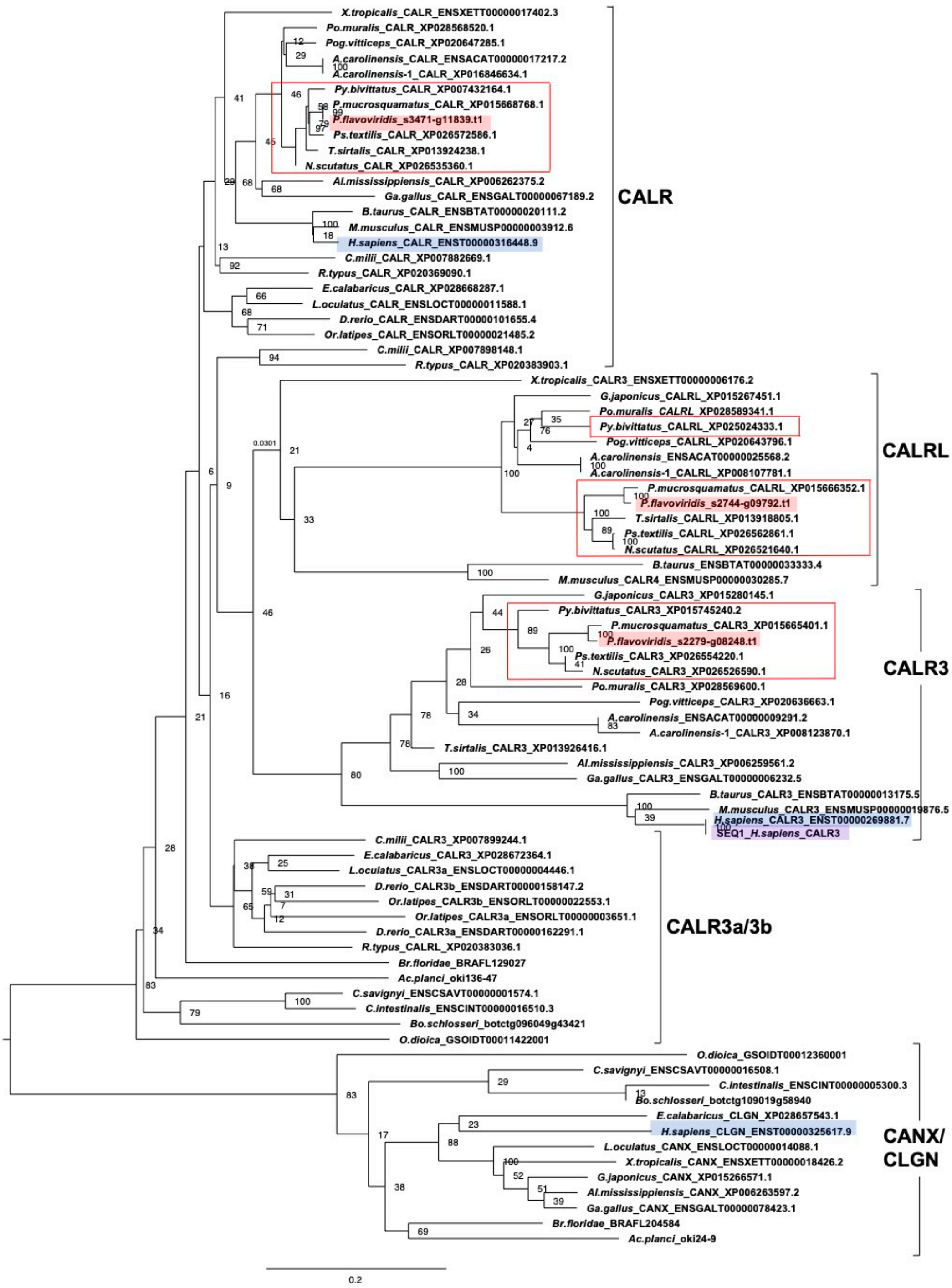

Figure S16. Molecular phylogenetic tree estimated using ORTHOSCOPE with human *CALR3* as the query sequence (highlighted in purple).

Sequences highlighted in blue indicate human orthologs. Those highlighted in pink represent habu (*P. flavoviridis*) orthologs, and those in red boxes represent other snake species. Values beside branches represent percentages of times that a node was supported in 100 bootstrap pseudoreplications implemented in ORTHOSCOPE. The scale bar indicates an evolutionary distance of 0.2 substitutions per position.

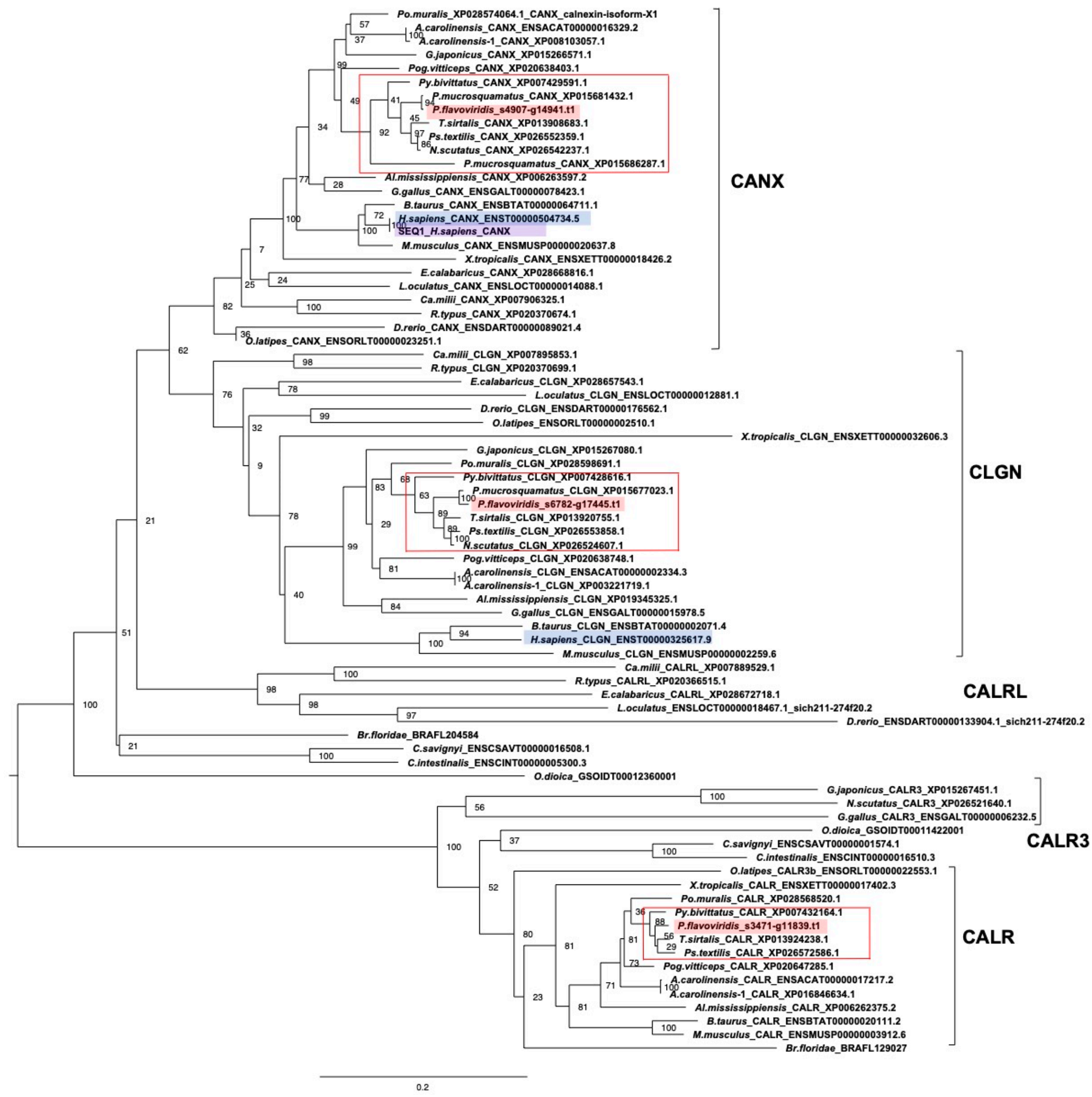

Figure S17. Molecular phylogenetic tree estimated using ORTHOSCOPE with human *CANX* as the query sequence (highlighted in purple).

Sequences highlighted in blue indicate human orthologs. Those highlighted in pink represent habu (*P. flavoviridis*) orthologs, and those in red boxes represent other snake species. Values beside branches represent percentages of times that a node was supported in 100 bootstrap pseudoreplications implemented in ORTHOSCOPE. The scale bar indicates an evolutionary distance of 0.2 substitutions per position.

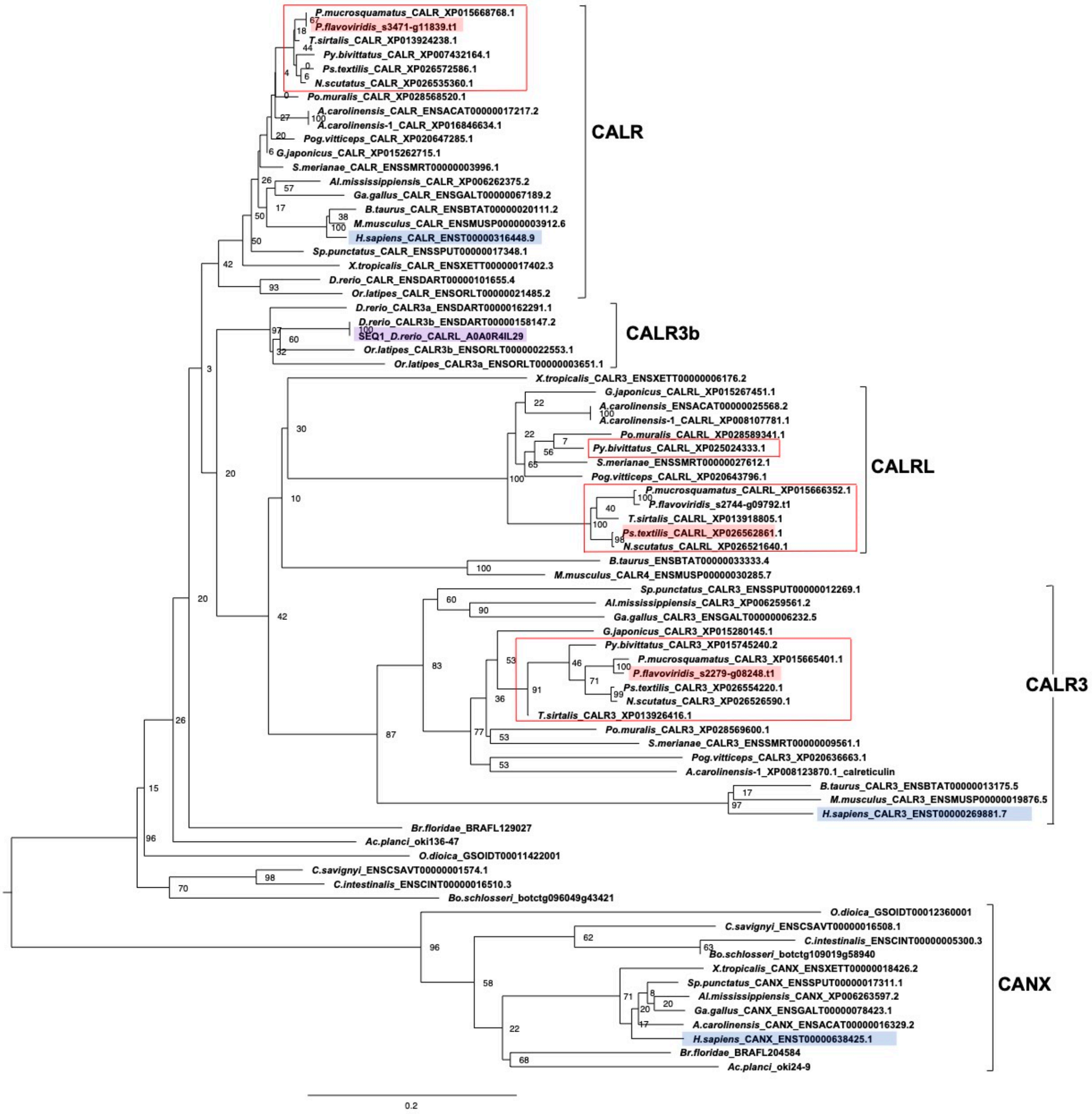

Figure S18. Molecular phylogenetic tree estimated using ORTHOSCOPE with *Danio rerio CALRL* as the query sequence (highlighted in purple).

Sequences highlighted in blue indicate human orthologs. Those highlighted in pink represent habu (*P. flavoviridis*) orthologs, and those in red boxes represent other snake species. Values beside branches represent percentages of times that a node was supported in 100 bootstrap pseudoreplications implemented in ORTHOSCOPE. The scale bar indicates an evolutionary distance of 0.2 substitutions per position.

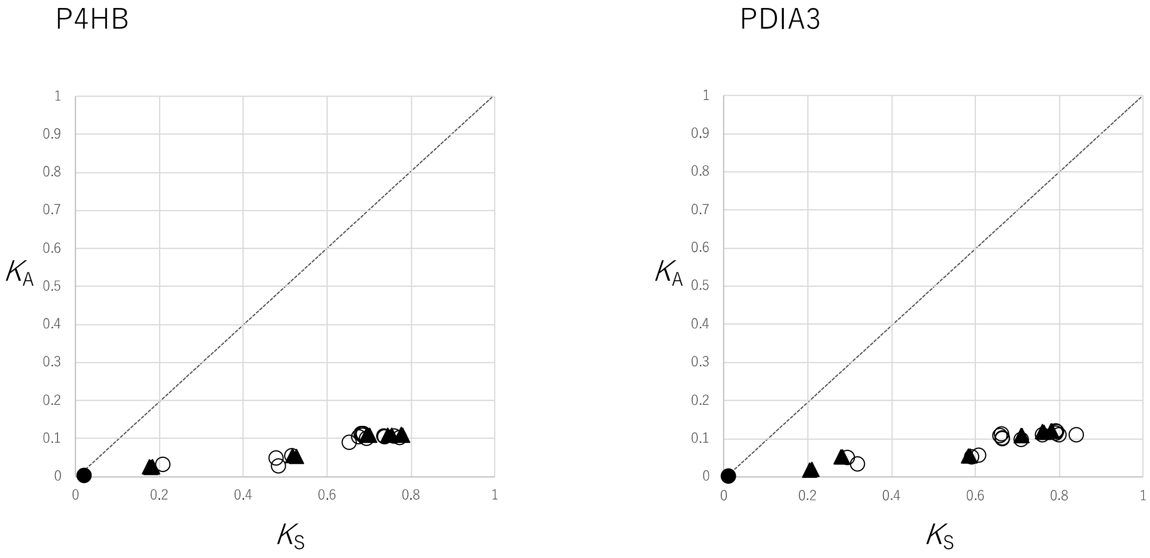

Figure S19. K_A/K_S plot for major venom protein modification candidate enzyme or chaperone genes, *P4HB* (left) and *PDIA3* (right).

K_A and K_S values were calculated by the Nei-Gojobori method for each pairwise genes: venomous snake vs venomous snake (●), venomous snake vs other animal (including human) (▲), and other animal vs other animal (○). Venomous snakes include *Protobothrops flavoviridis* and *P. mucrosquamatus*, and other animals include human, mouse, chicken, anole and non-venomous snakes (*Python bivittatus* and *Thamnophis sirtalis*).

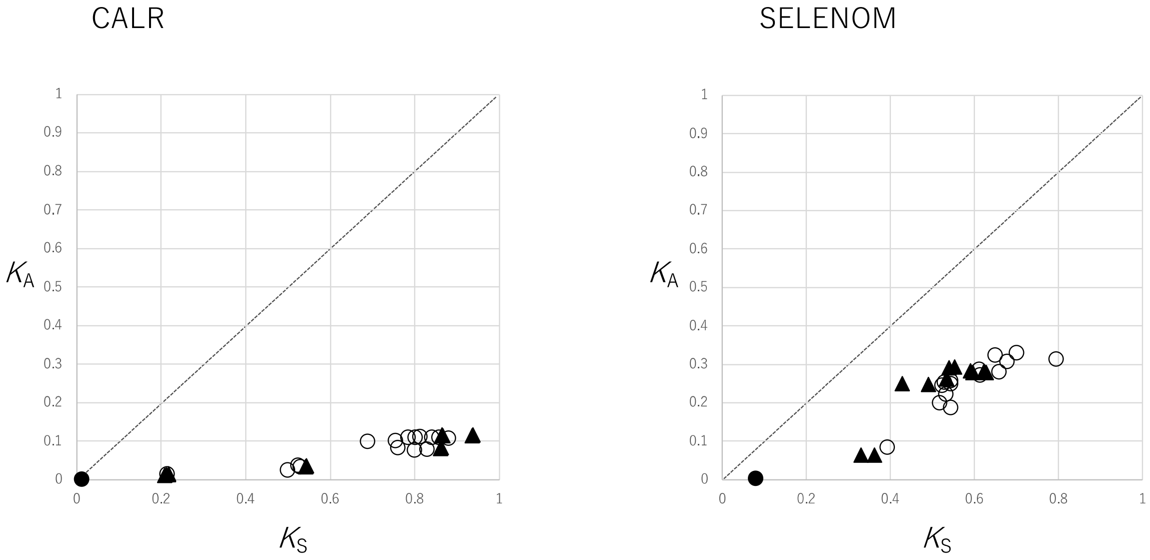

Figure S20. K_A/K_S plot for major venom protein modification candidate enzyme or chaperone genes, *CALR* (left) and *SELENOM* (right).

K_A and K_S values were calculated by the Nei-Gojobori method for each pairwise genes: venomous snake vs venomous snake (●), venomous snake vs other animals (including human) (▲), and other animals vs other animals (○). Venomous snakes include *Protobothrops flavoviridis* and *P. mucrosquamatus*, and other animals include human, mouse, chicken, anole and non-venomous snakes (*Python bivittatus* and *Thamnophis sirtalis*).
